# Slo1 Deficient Myoblast Exosomes‐Derived miR‐222‐3p Inhibits Osteogenic Differentiation via Targeting of STAT3

**DOI:** 10.1002/jcsm.70115

**Published:** 2025-12-08

**Authors:** Yonghui Wang, Xinrun Ma, Yang Chen, Chao Xia, Xue Lei, Junran Liu, Yunyi Jiang, Rang Xu, Yanhong Gao

**Affiliations:** ^1^ Department of Geriatrics, Xinhua Hospital Shanghai JiaoTong University School of Medicine Shanghai China; ^2^ Department of Geriatrics, Shanghai General Hospital Shanghai JiaoTong University School of Medicine Shanghai China; ^3^ Department of Endocrinology and Metabolism, Shanghai Ninth People's Hospital Shanghai JiaoTong University School of Medicine Shanghai China; ^4^ Scientific Research Center, Xinhua Hospital Shanghai JiaoTong University School of Medicine Shanghai China

**Keywords:** bone, exosomes, miR‐222‐3p, Slo1, STAT3

## Abstract

**Background:**

Previous research has shown that the conditional knockout of Slo1 in muscle leads to a reduction in muscle strength and inhibits myogenic differentiation. Exosomes are emerging as necessary mediators of the crosstalk between muscle and bones. The present study focused on the communication between muscle and bone to investigate the effects and mechanisms of skeletal muscle‐specific knockout of Slo1 on bone mass and bone metabolism.

**Methods:**

Myf5‐Cre mice were mated with Slo1^flox/flox^ mice to construct skeletal muscle‐specific Slo1 knockout mice (CKO mice). The legs of the Slo1 CKO mice were isolated, and a series of examinations were conducted to monitor bone mineral density (BMD) and bone microstructure. Exosomes were extracted from C2C12‐shNC and C2C12‐shSlo1 cells and subjected to transmission electron microscopy (TEM), nanoparticle tracking analysis (NTA) and Western blotting (WB). The osteogenic effect of EXO‐shSlo1 was examined in vitro and in vivo by Alkaline phosphatase (ALP) staining, q‐PCR, WB and microCT. RNA sequencing (RNA‐seq) analysis of EXO‐shNC and EXO‐shSlo1 was used to identify differentially expressed microRNAs. Mimics and inhibitors of miR‐222‐3p were transfected into MC3T3‐E1 cells to induce differentiation. The predicted targets of miR‐222‐3p were examined with Luciferase, qPCR, and WB.

**Results:**

In Slo1 CKO mice, the bone mass decreased, and the microstructure was disrupted. TEM, NTA and WB assays showed that Slo1‐silenced C2C12 cells secreted exosomes and localized to MC3T3‐E1 cells and bone tissue through the circulation. EXO‐shSlo1 inhibited osteogenesis both in vivo and in vitro, as demonstrated by the decreased ALP activity (~40%, *p* < 0.05), osteogenic marker expression (~30%, *p* < 0.05), mineral deposition in osteoblasts and BMD. qPCR was performed to confirm the exosome RNA‐seq results, which indicated that miR‐222‐3p was increased by three times in the EXO‐shSlo1 group compared with the EXO‐shNC group. Transfection of mimics or inhibitors of miR‐222‐3p in MC3T3‐E1 cells inhibited or improved osteogenic differentiation. Luciferase reporter assays revealed that miR‐222‐3p targets STAT3. The mRNA and protein level of STAT3 was affected by miR‐222‐3p. Through inhibiting miR‐222‐3p or upregulating STAT3, the EXO‐shSlo1‐mediated osteogenic inhibition of MC3T3‐E1 cells was ameliorated, as indicated by increased ALP activity and osteogenic marker expression.

**Conclusion:**

Slo1 deficiency in muscle results in decreased bone mass mediated by exosomal miR‐222‐3p. MiR‐222‐3p binds and inhibits the transcription of STAT3 to suppress the differentiation of osteoblasts. Slo1‐silenced myoblasts‐derived exosomal miR‐222‐3p inhibit osteogenesis and cause bone loss through the miR‐222‐3p/STAT3 pathway.

## 1 Introduction

Osteosarcopenia, an age‐related syndrome, is characterized by the co‐occurrence of osteoporosis (low bone density) and sarcopenia (reduced muscle mass, strength and function), yet remains frequently underdiagnosed [1, 2]. Among community‐dwelling older adults (≥ 65 years), variations across racial/ethnic groups and genders contribute to different prevalence of osteosarcopenia ranges from 5% to 37%, with higher rates observed in women (25.5%–82.6%) compared to men (16.4%–32%) [2]. The dual musculoskeletal deficits heighten fall and fracture risks, while mortality rates at 1 year are 1.8‐fold greater compared to healthy individuals or those with single conditions [3, 4]. As the global population ages, the growing incidence of osteosarcopenia is placing a substantial financial burden on healthcare systems and increasing the demand for long‐term care [5]. Although treatments for osteoporosis and sarcopenia have been developed, the mechanisms underlying osteosarcopenia remain poorly understood, limiting the availability of targeted therapies.

The Slo1 channel, also known as BK or MaxiK, exhibits large single‐channel conductance and is regulated by both intracellular Ca^2+^ and membrane voltage [6]. Slo1 plays a critical role in various diseases, including hypertension, type 1 and type 2 diabetes mellitus, obesity and autism [7, 8, 9, 10, 11]. In the musculoskeletal system, Slo1 influences muscle contraction, and knockout models have demonstrated reduced motor function and decreased bone mineral density [12, 13]. Specifically, muscle‐specific Slo1 deletion leads to muscular dysfunction; for example, Slo1 deletion in rats resulted in impaired skeletal and cardiac muscle function but enhanced smooth muscle contraction [14]. Our previous research found that conditional Slo1 knockout in muscle reduced running endurance and strength by 30% and inhibited myoblast differentiation, confirming the role of Slo1 deficiency in movement anomalies [15]. Collectively, Slo1 is essential for maintaining musculoskeletal health.

Bone and muscle are closely interdependent structures that are both spatially adjacent and functionally connected. They communicate through various mechanisms, including mechanical loading, secretion of signalling factors and exosome‐mediated delivery [16]. Reduced mechanical stimuli from muscle can lead to a gradual decline in bone mass [17]. For example, astronauts experience reductions in both muscle strength and bone mass during space travel, which resembles the bone loss seen in postmenopausal osteoporosis [18]. Recent studies have suggested that bone and muscle interact with each other through the secretion of myostatin, IGF‐1, irisin and extracellular vesicles [19, 20, 21, 22, 23]. For instance, exercise‐induced increases in IGF‐1 levels promote bone formation, while irisin, also released during exercise, enhances osteoblast differentiation through the Wnt/β‐catenin pathway [24]. Muscle cells also communicate with other cells through exosome secretion [25, 26]. In a hydrogen peroxide‐induced senescence model, myoblast‐derived exosomes exhibited high levels of miR‐34a, which is implicated in cellular senescence across multiple cell types. miR‐34a has been shown to inhibit osteoblast viability, suggesting its role in bone‐muscle crosstalk during aging [27, 28]. While the effects of mechanical loading and growth factor‐mediated bone loss have been extensively studied and recognized, the role of exosomal mechanisms remains underexplored. Extracellular vesicles are now recognized as key players in bone‐muscle crosstalk, which is not only involved in the mechanism but also therapy of musculoskeletal disorder. Importantly, miRNAs are known to play critical roles in bone remodelling and muscle‐bone crosstalk, making them ideal candidates for investigating the molecular mechanisms underlying exosome‐mediated bone loss. Additionally, miRNAs are highly stable within exosomes and can be efficiently transferred to target cells, where they exert significant regulatory effects. The decreased grip strength and running duration in CKO mice, despite no reduction in muscle mass, reflect impaired muscle function, which could potentially impact bone health. Given the limited research on gene‐related osteosarcopenia, the CKO mice we have established may serve as a viable model for studying this condition. Consequently, we investigate exosome‐mediated bone‐muscle crosstalk in our newly found CKO model, providing new insight in osteosarcopenia.

The current study demonstrated that bone mass is significantly reduced in the Slo1 conditional knockout (CKO) muscle atrophy model. Additionally, the findings suggest that myoblast‐derived exosomes can be internalized by bone, where they localize and inhibit osteoblast differentiation, ultimately leading to bone loss in mice. RNA sequencing (RNA‐seq) analysis revealed an enrichment of specific miRNAs in exosomes derived from Slo1 CKO myoblasts. Notably, qPCR analysis confirmed that miR‐222‐3p expression is elevated in both exosomes derived from Slo1‐deficient primary myoblasts and Slo1 CKO myoblasts. Furthermore, the results indicate that exosomal miR‐222‐3p inhibits osteoblast differentiation by directly suppressing the expression of STAT3.

## 2 Materials and Methods

### 2.1 Generation and Maintenance of Transgenic Mice

The skeletal conditional knockout (CKO) Slo1 mice were previously described in our study [15]. In brief, the Slo1^flox/flox^ mouse line was crossed with Myf5‐Cre mice (Jackson Laboratories) to generate the CKO mice. Mice were housed in specific pathogen‐free (SPF) facilities on a 12‐h light cycle at 22°C ± 2°C with standard chow and free access to tap water. All mice were bred on the background of the C57BL/6 line. Genotyping of transgenic mice was performed via PCR as previously described. The CKO and WT mice were sacrificed at 6–8 weeks age. We pooled four male and three female genders together to enhancing statistical power and broaden the application in all gender. All mentioned animal work was approved by the Ethics Committee of Xinhua Hospital Affiliated with Shanghai Jiao Tong University School of Medicine.

### 2.2 OVX Model

Healthy female C57BL/6 mice (8 ~ 12 weeks, 20 ~ 25 g) were housed in SPF conditions. After a 7‐day acclimatization, animals were anaesthetized via intraperitoneal injection of 1% pentobarbital sodium (40 mg/kg). Surgical areas (dorsal or ventral regions) were shaved and disinfected with alternating iodine and 75% ethanol. Bilateral longitudinal incisions (1.5 cm) were made parallel to the spine at the L1‐L3 level. Ovaries, identified as pink granular structures within the adipose tissue, were gently exteriorized. The oviducts near the uterine horns were double‐ligated with 4‐0 Vicryl sutures, and ovaries were excised. Haemostasis was achieved via electrocautery before closing muscle and skin layers. Animals were placed on a 37°C heating pad until full recovery. Prophylactic antibiotics (penicillin, 50 000 IU/kg; or enrofloxacin, 10 mg/kg) were administered subcutaneously for 3 days. Analgesia (ibuprofen, 0.1 mg/mL in drinking water) was provided for 24 h. Incision healing, activity and body weight were monitored daily; animals with complications were excluded.

### 2.3 Microcomputed Tomography (micro‐CT)

As previously described [16], the ethanol‐fixed tibias at 6 ~ 8 weeks of age were analysed to obtain quantitative data on the trabecular and cortical bone via a micro‐CT system (R_mCT; Rigaku Corporation, Tokyo, Japan) at a resolution of 10 μm/voxel. The diaphyseal part of the tibias with a thickness of 2 mm was prepared, and the structure of cortical bone was analysed by a SkyScan 1272 (Bruker, Billerica, MA, USA) at a resolution of 0.5 μm/voxel [29].

### 2.4 Histology

The bones were separated from the mice and fixed with 4% PFA at 4°C overnight. After water washes, the bone was decalcified with EDTA. The samples were embedded in paraffin, sectioned (5 μm thickness) and sequentially deparaffinized in xylene and graded alcohols, followed by hydration. Subsequent staining protocols were performed as follows:

H&E: Sections were stained with haematoxylin for 5–10 min, differentiated in 1% hydrochloric acid–alcohol solution, rinsed in water and counterstained with eosin for 1–3 min.

Masson's Trichrome: Tissues were first stained with Masson's red for 5–10 min, followed by Picrosirius solution.

Alkaline phosphatase (ALP): Sections were incubated with ALP staining solution (containing NaCl, MgCl_2_, etc.) at 37°C for 30–60 min to visualize enzyme activity.

Tartrate‐resistant acid phosphatase (TRAP): Samples were incubated with TRAP staining solution (including NaCl, MgCl_2_, tartaric acid) at 37°C for 30–60 min until osteoclasts appeared red.

Post‐staining, HE‐ and Masson‐stained slides underwent alcohol dehydration, xylene clearing and cover slipping, while ALP‐ and TRAP‐stained sections were directly rinsed, cleared and mounted. The quantification of H&E and Masson's Trichrome staining was performed with ImageJ (v1.53). The thresholding was applied to identify positive areas, followed by automated area measurement. A fixed intensity threshold was applied across all images prior to measurement. Blinding was not performed as the analysis was fully automated based on pre‐defined parameters.

### 2.5 Exosome Extraction

Prior to exosome isolation, cells were cultured for 48 h in complete medium supplemented with exosome‐depleted FBS (prepared by ultracentrifugation at 100 000 ×*g* for 16 h at 4°C). The cell culture medium was collected and centrifuged at 2000 ×*g* for 30 min to remove cells and debris. The cell‐free medium was transferred to a new tube and mixed well with an equal volume of Total Exosome Isolation Reagent (Thermo Fisher Scientific, USA). The mixture was incubated at 4°C overnight and then centrifuged at 10 000 ×*g* for 1 h at 4°C. The pellets were suspended in PBS and stored at −80°C.

### 2.6 Characteristics of Exosomes

The exosomes were characterized by transmission electron microscopy (TEM), nanoparticle tracking analysis (NTA), and Western blotting (WB). The exosome suspension (20 μL) was placed on carbon mesh and incubated for 20 min at room temperature. The excess exosome suspension was carefully blotted with filter paper, 20 μL of 2% phosphotungstic acid was added to the carbon mesh, and the mixture was incubated for 20 s. The excess phosphotungstic acid was carefully removed with filter paper, and the carbon mesh was placed in a glass dish lined with filter paper. Images were then acquired (JEOL, JEM‐1400). For NTA, the sample was washed with deionized water, and the instrument was calibrated with polystyrene microspheres (110 nm). After washing and dilution with 1X PBS buffer, the samples were injected for detection (Metrix, ZetaView PMX 110). Exosome concentrations were determined using bicinchoninic acid (BCA) assay and subsequently normalized to a standardized concentration of 100 μg/mL.

### 2.7 Exosome Labelling and Cellular Uptake

Exosomes were incubated with PKH26 cells for 30 min at room temperature and washed with PBS. Then, 5 μg of labelled exosomes was added to MC3T3‐E1 cells for 6 h at 37°C. Nuclei were stained with DAPI after fixation with 4% PFA. Images were taken under a confocal microscope.

### 2.8 Ex Vivo Imaging of Fluorescently Labelled Exosomes

PBS and 100 μg of PKH26‐labelled exosomes were injected into C57BL/6 mice via the tail vein. The mice were sacrificed at 4 h, 8 h post‐injection. The main organs, including the heart, spleen, lung, kidney, liver, and bones, were separated and imaged using a VISQUE Compact Small Animal Fluorescence & Bioluminescence Imaging System (Vieworks Inc., South Korea).

### 2.9 Cell Culture

MC3T3‐E1, HEK293, and C2C12 cells were purchased from Shanghai Institutes for Biological Sciences (Shanghai, China) and cultured in Dulbecco's modified Eagle's medium (DMEM) supplemented with 10% foetal bovine serum (FBS), 100 U/mL penicillin and 100 mg/mL streptomycin. The cells were cultured in a 5% CO_2_ incubator at 37°C. For osteogenic induction, DMEM supplemented with 10% FBS, 100 U/mL penicillin, 100 mg/mL streptomycin, 50 μg/mL ascorbic acid, 100 nM dexamethasone and 10 mM β‐glycerophosphate was used. A standardized concentration of 2.5 μg/mL exosomes was applied per well in vitro. During osteogenic induction, media were refreshed every 48 h, and exosomes were replenished at each media change to maintain consistent bioactivity.

Primary myoblasts were isolated from murine hindlimb skeletal muscles using the following protocol: Muscle tissues were minced into 1 ~ 2 mm^3^ fragments, digested with 0.2% collagenase II (37°C, 120 rpm agitation for 45 min), filtered through a 100 μm strainer and centrifuged at 300 ×*g* for 5 min. Pelleted cells were resuspended in Ham's F‐10 medium supplemented with 20% FBS, 1% penicillin–streptomycin, and 10 ng/mL bFGF. Fibroblasts were removed via 15‐min differential adhesion, and purified myoblasts were cultured on collagen‐coated plates until reaching 80% confluency. The primary myoblasts cells were used to extract the exosomes same as C2C12 before passage 5.

### 2.10 Cell Transfection

MiR‐222‐3p mimics, miR‐222‐3p inhibitors and negative controls were purchased from RiboBio (China) and used to transfect cells according to the manufacturer's instructions. Briefly, the cells (Passage 3–10) were cultured in 6‐well or 12‐well culture plates and transfected with 50 nM miR‐222‐3p mimics, miR‐222‐3p inhibitors or negative controls using Hieff Trans Liposomal Transfection Reagent (Yeason, China) for 24 h. Exosomes or an equal volume of PBS was then added to the medium. Subsequent experiments were performed after 24 h.

The overexpression of STAT3 was achieved in MC3T3‐E1 cells by plasmid transfection. We commissioned Shanghai GenePharma Co. Ltd. to synthesize a recombinant plasmid using the PEX‐3 vector. When MC3T3‐E1 cells reached to 50% ~ 70% confluence, 5 μg of PEX‐1 (as control) or PEX‐3‐STAT3 were transfected into MC3T3‐E1 cells using Hieff Trans Liposomal Transfection Reagent (Yeason, China). The medium was replaced 3 days post‐transfection to proceed with subsequent experiments.

### 2.11 Adenovirus Design and Construction

The adenovirus vector expressing short hairpin RNA (shRNA) targeting the sequence of Slo1 gene (GCTTGAGGCTCTGTTCAA, GCACTTACGTACTGGGAAT, GCATGTGGTGGGCTTTCTT) and negative control (TTCTCCGAACGTGTCACGT) were synthesized and cloned into GV290 (hU6‐MCS‐CMV‐Puromycin) vector with AgeI and EcoRI sites (purchased from Shanghai Genechem Co. Ltd.), recombinant vector was detected by DNA sequencing.

The shuttle plasmid was recombined with pBHGlox_ΔE1,3Cre plasmid (Microbix) in HEK293A cells. Once an extensive cytopathic effect was observed, the virus was harvested from HEK293A cells using three freeze–thaw cycles. Single clones were selected by serial dilution and amplified by serial infection, followed using an Adeno‐X Virus Purification Kit (Takara) to produce concentrated and the adenovirus titre in plaque‐forming units (pfu) was determined by a plaque formation assay in HEK293 cells. Three sequences were distinctly inhibited Slo1 expression, we chose AdshSlo1–2 in the following tests.

### 2.12 Alkaline Phosphatase (ALP) Staining and Activity

Exosomes were applied to MC3T3‐E1 cells, and osteogenesis was induced with osteogenic medium. The medium was changed and the exosomes was added every 3 days. After 7 days of induction, the cells were fixed with 4% PFA and lysed with RIPA buffer. ALP activity was measured using an ALP assay kit (Beyotime, China) and normalized to the protein concentration (Beyotime, China). The fixed cells were stained with a BCIP/NBT alkaline phosphatase staining kit (Beyotime, China).

### 2.13 Alizarin Red Staining

After 14 days of osteogenic induction with osteogenic medium, the cells were fixed with 4% PFA at room temperature for 20 min and washed three times with PBS. The fixed cells were stained with 2% Alizarin Red S (ARS) solution (Sigma‐Aldrich, USA) for 30 min at room temperature and then washed thoroughly with PBS. Mineral nodules were observed by light microscopy (Leica DMI 3000B, Germany).

### 2.14 Quantitative Real‐Time PCR

Total RNA was extracted from cells and tissue with Trizol as previously reported [30] (TaKaRa Biotechnology, Japan). cDNA was synthesized from 1 μg of total RNA using PrimeScript RT Master Mix (TaKaRa Biotechnology, Japan). qPCR analysis was performed with 2× SYBR master mix (Yeason, China). MiRNA was reverse transcribed to cDNA with a miDETECT A Track miRNA qPCR Starter Kit (RiboBio, China) according to the manufacturer's instructions. The miRNA‐specific forward primer and the universal reverse primer were designed by RiboBio (RiboBio, China). U6 small nuclear RNA was used to normalize the results. The qPCR primers used are listed in Table S1.

### 2.15 Western Blot Analysis

Cells were lysed in cold RIPA buffer supplemented with protease and phosphatase inhibitors. Bone was lysed with the same buffer and smashed with stainless steel grinding balls. The concentration of the sample was measured with a BCA protein assay kit (Beyotime, China) following the manufacturer's instructions. The samples were loaded onto SDS‐polyacrylamide gels, and proteins were transferred to PVDF membranes. The membranes were incubated with primary antibody (CD81, TSG101, GAPDH, ALP, OPN, Runx2, Tubulin, STAT3, Sigma, 1:1000) at 4°C overnight, followed by incubation with an HRP‐conjugated secondary antibody (HRP‐labelled Goat Anti‐Rabbit IgG, sigma, 1:2000; HRP‐labelled Goat Anti‐Mouse IgG, sigma, 1:2000) at 37°C for 1 h. The immunoreactive bands were visualized using an enhanced chemiluminescence reagent (Thermo Fisher Scientific, USA) and imaged with a ChemiDocTM MP Imaging System (Bio‐Rad, USA).

Protein bands were quantified using ImageJ (v1.53). Raw TIFF images were converted to 8‐bit grayscale. Rectangular ROIs of identical size were drawn around target bands and adjacent background areas. Background subtraction was performed using the Rolling Ball algorithm (radius = 50 pixels). Integrated density values (IDV) were measured for each band, Target protein levels were normalized to loading controls (e.g., α‐Tubulin) within the same lane. Minimum triplicate blots were analysed per condition.

### 2.16 Dual Luciferase Reporter Assay

Mouse wild‐type or mutant STAT3 3′ untranslated regions (UTRs) were chemically synthesized by RiboBio (Guangzhou, China), cloned and inserted into pmirGLO luciferase reporter plasmids. The recombinant plasmids used were pmirGLO‐STAT3 3′UTR‐WT and pmirGLO‐STAT3 3′UTR‐mut. HEK293T cells were plated in 6‐well plates and allowed to reach 70% confluence. The cells were then cotransfected with 2 μg of pmirGLO‐STAT3 3′UTR‐WT/pmirGLO‐STAT3 3′UTR‐mut plasmids with either the miR‐222‐3a mimic/inhibitor or the negative control (NC) with Hieff Trans Liposomal Transfection Reagent (Yeason, China). At 48 h post‐transfection, firefly and Renilla luciferase activities were examined with a dual luciferase assay system (Promega, USA).

### 2.17 RNA‐Seq

The experimental workflow began with RNA quality assessment using 1% agarose gels, spectrophotometry (NanoPhotometer) and the Agilent Bioanalyzer 2100 system to evaluate degradation, purity, concentration and integrity. Libraries were prepared with NEBNext kits, involving adapter ligation (3′ and 5′ ends), reverse transcription, PCR amplification and size selection (140–160 bp fragments) before Illumina Hiseq sequencing (50 bp single‐end reads). For small RNA analysis, data processing included quality filtering (custom scripts), genome alignment via Bowtie (zero mismatches) and annotation of known miRNAs (miRBase/miRDeep2) and novel miRNAs (miREvo) after removing non‐target RNAs (RepeatMasker/Rfam). Hierarchical annotation prioritized miRNA > rRNA > tRNA, followed by seed region editing analysis, miRNA family classification (miFam.dat/Rfam) and target prediction (psRobot/miRanda). Differential expression analysis employed DESeq (with replicates) or DEGseq (without replicates) with TPM normalization, and functional enrichment (GO/KEGG) was conducted using GOseq/KOBAS.

RNA‐Seq bioinformatics workflows included raw data trimming (Trimmomatic), alignment (HISAT2) and quantification at gene (Cufflinks/FPKM) and transcript levels (Bowtie/eXpress). Differentially expressed genes/transcripts (DEGs) were identified (DESeq, fold change > 2/< 0.5, *p* < 0.05), clustered and subjected to GO/KEGG enrichment via hypergeometric tests. Transcriptome assembly (StringTie), novel transcript identification (Cuffcompare), alternative splicing analysis (ASprofile) and variant calling (SNPs/INDELs via samtools/bcftools) with functional annotation (SupEff) were integrated. Both workflows utilized tools like HTSeq‐count and DESeq for robust analysis of gene/isoform expression, miRNA dynamics and genomic variations, ensuring comprehensive insights into regulatory mechanisms and structural variations.

### 2.18 Statistics

All the data are expressed as the means ± standard deviations (SDs). Student's *t* test (for comparisons between two groups) and one‐way ANOVA (for comparisons among more than two groups), followed by the Bonferroni post hoc correction, were used for the statistical analysis. The statistical analysis was conducted using GraphPad software. *p* < 0.05 was considered significant.

## 3 Results

### 3.1 Slo1 Knockout in Skeletal Muscle Causes Bone Loss

**FIGURE 1:**
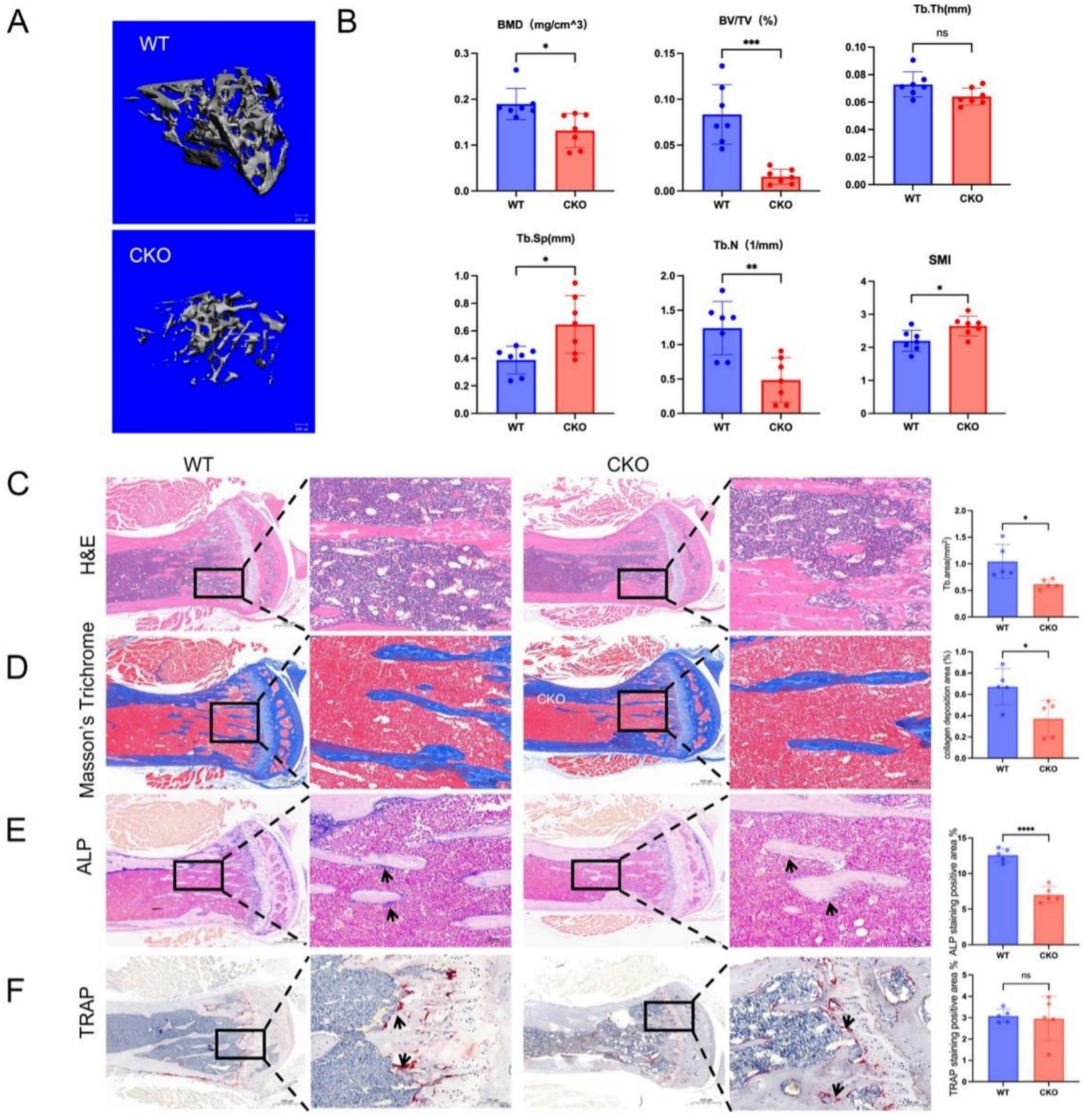
Slo1 CKO leads to bone loss and deterioration of the microstructure. (A) 3D reconstruction of microCT images of tibias from WT and CKO mice (*n* = 7, four male and three female mice). (B) Quantitative analysis of the bone mineral density (BMD), bone volume per total volume (BV/TV), structure model index (SMI), trabecular number (Tb. N), trabecular thickness (Tb. Th) and trabecular separation (Tb. Sp) of WT and CKO tibias. (C) Representative H&E images of femurs from WT and CKO mice and the trabecular area in H&E staining. (D) Representative Manson's Trichrome staining images of femurs from WT and CKO mice and the collagen deposition area (%) in Manson's Trichrome staining. (E) Representative images of ALP histological staining of femurs from WT and CKO mice. The analysis of the positive area of ALP staining. (F) Representative images of TRAP histological staining of femurs from WT and CKO mice. The analysis of the positive area of TRAP staining. (*n* = 5, three male and two female mice); *p < 0.05 and **p < 0.01. ALP (alkaline phosphatase, a marker of osteoblast activity) and TRAP (tartrate‐resistant acid phosphatase, a marker of osteoclast activity).

Previous research has shown that knockout of Slo1 in skeletal muscle results in reduced endurance and strength, making it a suitable model for studying sarcopenia [15]. Deficiency of Slo1 in osteoblasts leads to bone loss; we further evaluate the Slo1 expression in CKO bones. As shown in Figure S1A,B, the mRNA and protein level of Slo1 are not affected by muscular knockout Slo1. Given that muscular contraction is a key mechanical stimulator of osteogenesis in osteoblasts and bone marrow stromal cells (BMSCs), we assessed tibial bone mass in Slo1 conditional knockout (CKO) mice using micro‐CT. As demonstrated in Figure 1A,B, trabecular bone density was markedly lower in CKO mice compared to wild‐type (WT) mice at 6 ~ 8 weeks of age (pooled four male and three female mice). Bone morphometric parameters, including bone mineral density (BMD), bone volume fraction (BV/TV), and trabecular number (Tb. N), trabecular thickness (Tb. Th), and trabecular separation (Tb. Sp) were significantly reduced in the tibia of CKO mice, while the structure model index (SMI) was increased. Additionally, cortical bone scans revealed no notable differences in cortical bone structure, the bone area/tissue area (BA/TA) and cortical thickness (Ct. Th), between the groups (Figure S1C,D). Additionally, the sex‐based analysis of micro‐CT analysis showed that the BMD, BV/TV, Tb. N were decreased in both male and female of CKO mice comparing with WT mice. The other trabecular indexes, Tb. Th, Tb. Sp and SMI, and cortical indexes, BA/TA and Ct. Th were not altered in both groups showed no significant difference in CKO and WT mice (Figure S1E). Histological analysis of the distal femurs using H&E and Masson's Trichrome staining showed less trabecular bone and collagen deposition in CKO mice femurs (Figure 1C,D). ALP staining was significantly stronger in the WT group compared to the CKO group (Figure 1E), indicating higher osteoblasts activity reflecting increased bone formation. In contrast, TRAP staining, which is used to identify osteoclast activity reflecting bone resorption, showed no significant difference between the two groups (Figure 1F).

### 3.2 Exosomes Are Secreted From Slo1‐Deficient or Wildtype Myoblasts and Target Bones

**FIGURE 2:**
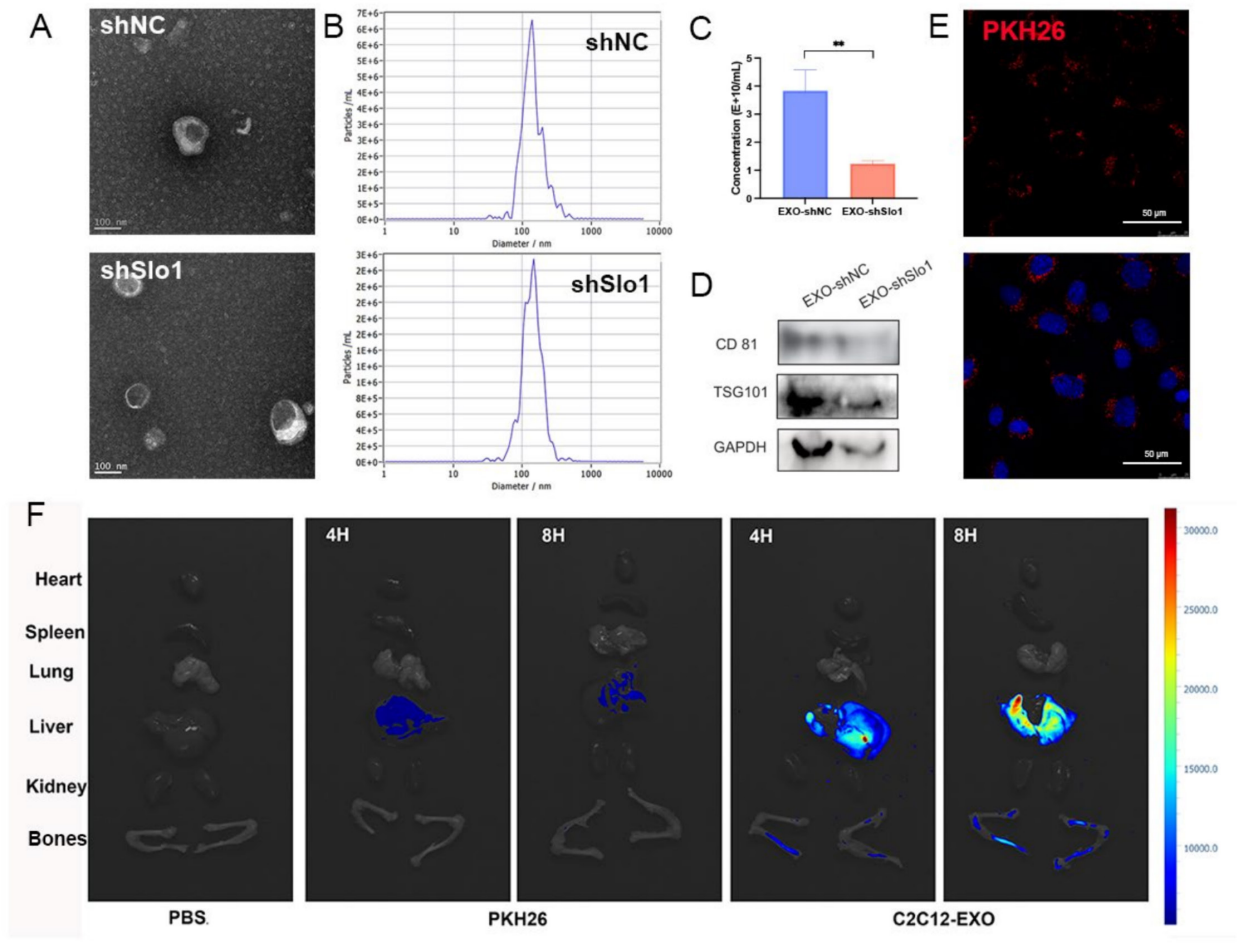
Myoblasts excrete exosomes and target osteoblasts and bones. (A) Representative transmission electron microscopy images of EXO‐shNC and EXO‐shSlo1. (B) Representative NTA results of EXO‐shNC and EXO‐shSlo1 showing the distribution of particle diameters. (C) The concentration of exosomes detected by NTA. (D) The TSG101, CD81 and GAPDH exosomal markers were detected in EXO‐shNC and EXO‐shSlo1 by WB. (E) Confocal images of MC3T3‐E1 cells incubated with PKH26‐labelled exosomes for 6 h. The nuclei were stained with DAPI. (F) Biophotonic images of organs (heart, liver, lung, spleen, kidney and bone) from C57 mice injected with PBS, PKH26 or PKH26‐labelled exosomes via the tail vein for 4 or 8 h. (EXO‐shNC means the exosomes derived from C2C12 transfected with shNC; EXO‐shSlo1 means the exosomes extracted from C2C12 transfected with shSlo1).

Extracellular vesicles were isolated from the supernatant of C2C12 myoblasts infected with either shNC or shSlo1 adenovirus. The mRNA expression level of Slo1 decreased to 50% of that observed in the shNC group (Figure S2). Transmission electron microscopy (TEM) images revealed that both shNC and shSlo1 C2C12 cells secreted vesicles with a typical double‐layered morphology, with sizes approximately 150 nm (Figure 2A). Nanoparticle tracking analysis (NTA) showed that 96.5% of vesicles from shSlo1 C2C12 myoblasts were 137 nm in size, while 96.4% of vesicles from shNC C2C12 myoblasts were 134.6 nm (Figure 2B). The concentration of exosomes was higher in the EXO‐shNC group compared to the EXO‐shSlo1 group (Figure 2C). Western blot analysis confirmed the presence of the protein markers TSG101, CD81 and GAPDH in exosomes from both groups, indicating successful excretion of exosomes by both shNC and shSlo1 C2C12 cells (Figure 2D).

Exosomes exert specific functions on target cells or organs by being internalized and localized to particular sites. To trace the exosomes, they were labelled with PKH26, a red fluorescent membrane dye. As shown in Figure 2E, red puncta surrounding the DAPI‐stained nucleus were observed, indicating that exosomes were internalized into the cytoplasm. Furthermore, when PKH26‐labelled exosomes were injected into mice via the tail vein, fluorescence signals were detected in both the liver and bones (Figure 2F). These findings suggest that exosomes can be taken up by osteoblasts in vitro and by bones in vivo, highlighting the potential role of exosomes in skeletal system homeostasis.

### 3.3 Exosomes From Slo1‐Deficient Myoblasts Inhibit Osteoblast Differentiation In Vitro

**FIGURE 3:**
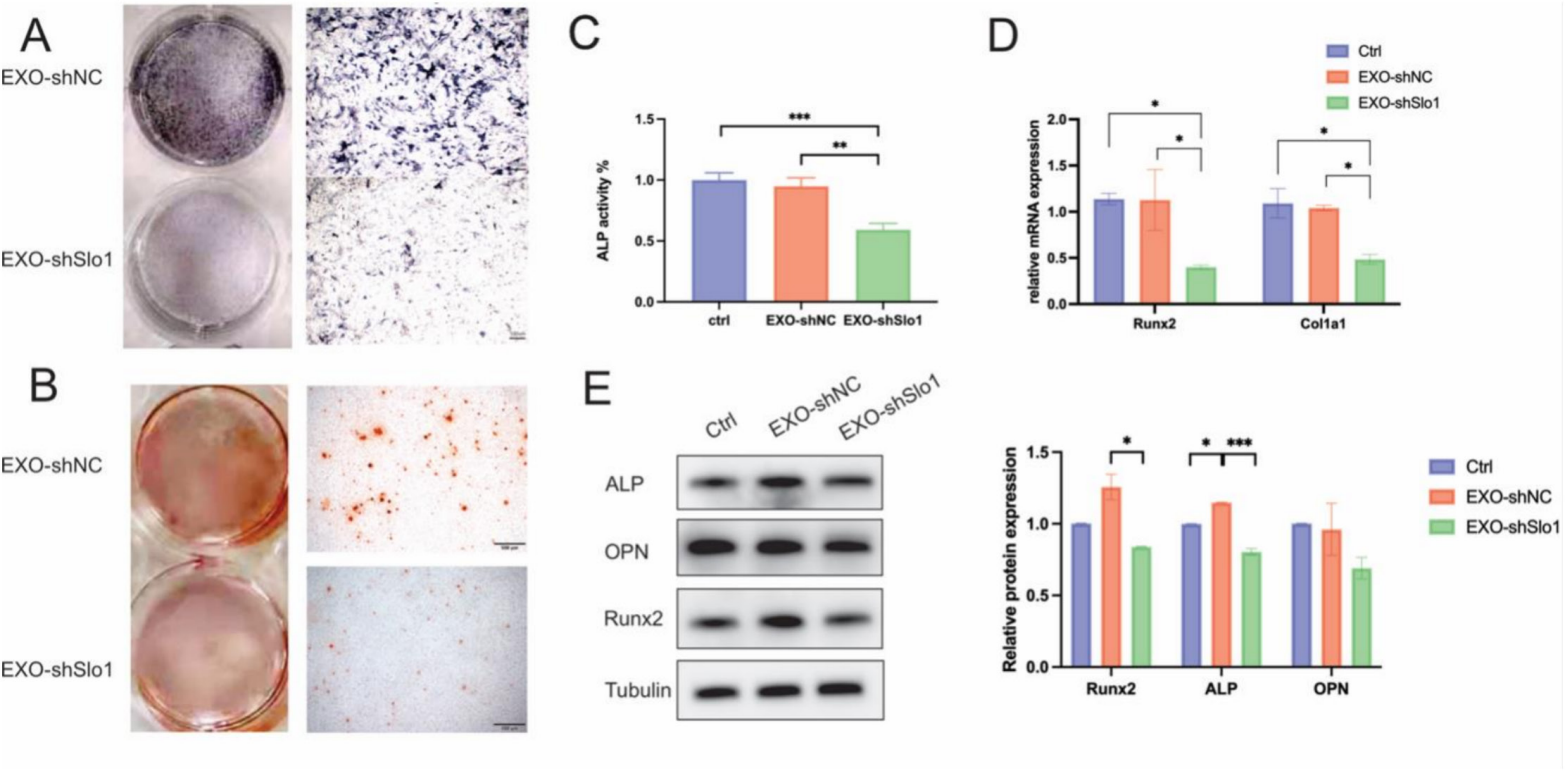
EXO‐shSlo1 induce osteogenic inhibition in MC3T3‐E1 cells. After treating MC3T3‐E1 cells with EXO‐shNC and EXO‐shSlo1, the cells were osteogenically induced for 7 days. (A) ALP staining was performed to assess osteogenic differentiation. (B) Representative ARS images of EXO‐shNC and EXO‐shSlo1treated MC3T3‐E1 cells after 21 days of osteogenic differentiation. ARS (Alizarin Red S, a dye used to detect calcium deposits as a marker of mineralization) staining results in bone tissues. (C) ALP activity was measured after 7 days of osteogenic induction and treatment with PBS, EXO‐shNC, or EXO‐shSlo1. (D) The mRNA expression levels of Runx2 and Col1a were assessed by q‐PCR. (E) The protein expression levels of the Runx2, ALP and OPN osteogenic markers were measured by WB in the Ctrl, EXO‐shNC and EXO‐shSlo1 groups. *n* = 3; *p < 0.05, **p < 0.01 and ***p < 0.001. (EXO‐shNC means the exosomes derived from C2C12 transfected with shNC; EXO‐shSlo1 means the exosomes extracted from C2C12 transfected with shSlo1).

To assess the biological effects of Slo1‐deficient myoblast‐derived exosomes on osteogenic differentiation, MC3T3‐E1 osteoblasts were treated with exosomes (2.5 μg/mL) for 72 h, and the early osteogenic marker ALP was evaluated by staining and activity assays. ALP staining revealed significant inhibition by EXO‐shSlo1 (Figure 3A). Mineralization in MC3T3‐E1 cells was also suppressed by EXO‐shSlo1 compared to EXO‐shNC as demonstrated in ARS staining, which is a marker of mineral deposition (Figure 3B). Quantitative ALP activity assays indicated a 40% reduction in ALP activity in the EXO‐shSlo1 group (Figure 3C). Q‐PCR analysis showed that the mRNA expression levels of osteogenic markers Runx2, and Col1a1 were reduced by approximately 50% in MC3T3‐E1osteoblasts treated with EXO‐shSlo1 (Figure 3D). Additionally, protein levels of Runx2, ALP and OPN were decreased in the EXO‐shSlo1 group (Figure 3E).

To further confirm the inhibitory effect of Slo1‐deficient myoblast‐derived exosomes on osteogenesis, exosomes were isolated from primary myoblasts of Slo1 knockout mice and used to treat MC3T3‐E1 osteoblasts. ALP staining and activity were decreased in the EXO‐CKO group (Figure S3A,B). Protein and mRNA levels of Runx2 and ALP were lower in EXO‐CKO compared to Ctrl and EXO‐WT (Figure S3C,D). These results suggest that exosomes from CKO myoblasts have a detrimental effect on osteoblast differentiation.

### 3.4 Exosomes From Slo1‐Deficient Myoblasts Cause Bone Loss

**FIGURE 4:**
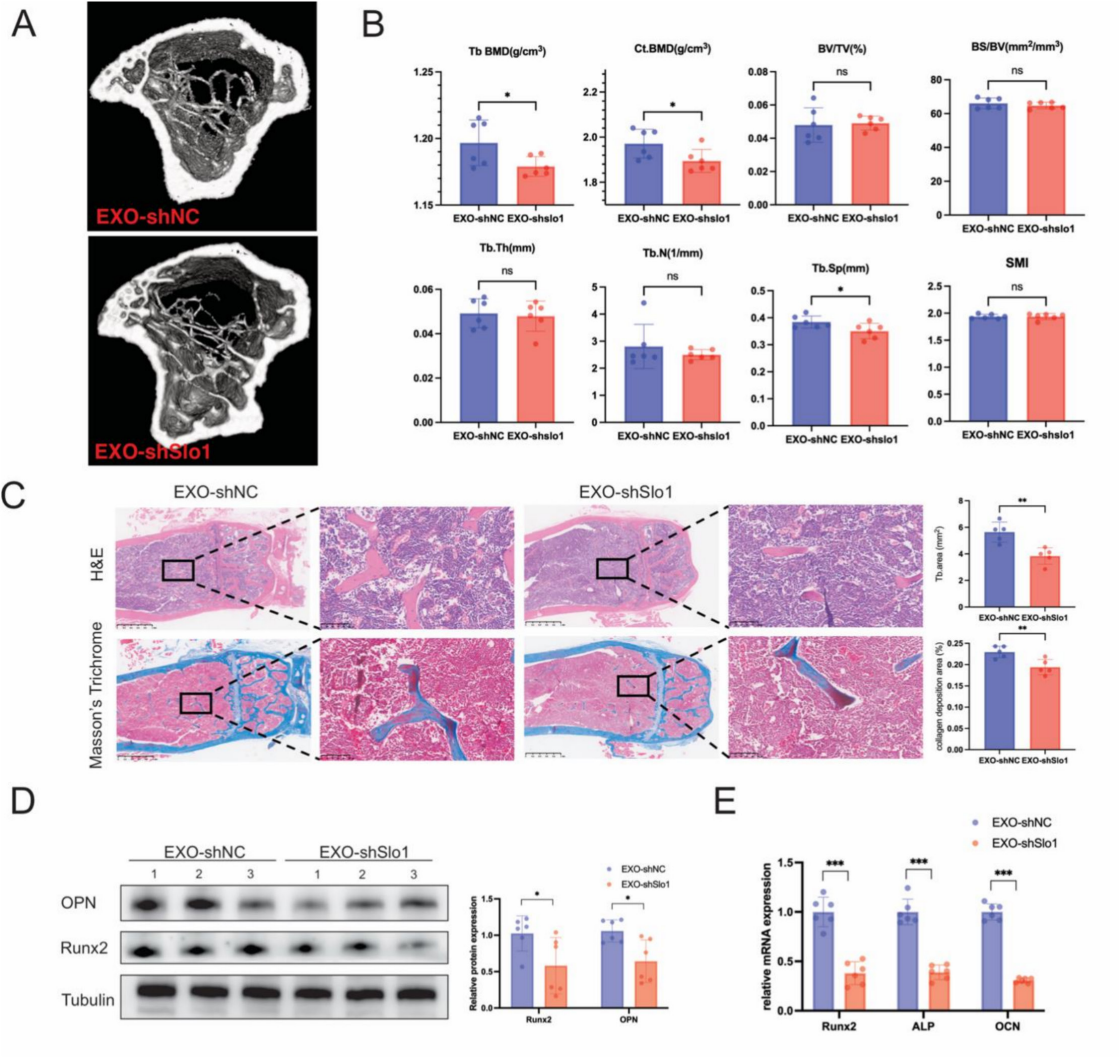
EXO‐shSlo1 inhibit bone formation in vivo. (A) Representative microCT images of tibias from EXO‐shNC‐ and EXO‐shSlo1‐injected mice (*n* = 6). (B) Quantitative analysis of trabecular BMD (Th. BMD), cortical BMD (Ct. BMD), BS/BV, BV/TV, Tb. Th, Tb. N, Tb. Sp and SMI. (C) Representative images of H&E staining (left panel) and Trichrome staining (right panel) of femurs from EXO‐shNC and EXO‐shSlo1 injected mice (*n* = 5). (D) Proteins were extracted from the legs of exosome‐treated mice. The protein levels of Runx2 and OPN were measured by WB. Tubulin was used as an internal reference (*n* = 6). (E) RNA was extracted from the legs of EXO‐shNC and EXO‐shSlo1 treated mice, and the mRNA expression levels of ALP, OCN and Col1a were measured by q‐PCR. *n* = 6; *p < 0.05, **p < 0.01 and ***p < 0.001. (EXO‐shNC means the exosomes derived from C2C12 transfected with shNC; EXO‐shSlo1 means the exosomes extracted from C2C12 transfected with shSlo1).

To further investigate the impact of exosomes from Slo1‐deficient myoblasts on osteoblasts, we performed an in vivo study in wild‐type mice. EXO‐shSlo1 and EXO‐shNC were injected via the tail vein twice a week (100 μg/mice/time) for 8 weeks. The legs were retrieved for micro‐CT and histological analyses. 3D reconstructed tibia images showed no obvious differences between trabecular and cortical bones (Figure 4A). However, detailed microstructural analysis revealed reductions in Ct BMD, Th BMD and BS/BV following EXO‐shSlo1 treatment (Figure 4B). H&E and Masson's Trichrome staining indicated that trabeculae were less and collagen deposition were less in the EXO‐shSlo1 group compared to the EXO‐shNC group (Figure 4C). Protein and RNA analyses from the bones revealed significant decreases in protein levels of Runx2 and OPN, as well as reduced RNA levels of ALP, OCN and Col1a1 in the EXO‐shSlo1 group (Figure 4D–E). These findings demonstrate that EXO‐shSlo1 induces bone loss in vivo.

To determine the molecular differences in MC3T3‐E1 cells treated with EXO‐shNC or EXO‐shSlo1, RNA sequencing was performed. A total of 62 differentially expressed genes (DEGs) were identified between the EXO‐shSlo1 and EXO‐shNC groups (|log2FC| > 1.5, *p* < 0.05) (Figure S4A). Gene Ontology (GO) enrichment analysis highlighted cell differentiation terms among the top‐ranked categories (Figure S4B). Kyoto Encyclopedia of Genes and Genomes (KEGG) enrichment analysis identified endocrine disease as an enriched pathway (Figure S4C). Gene set enrichment analysis (GSEA) revealed that EXO‐shSlo1 treatment negatively correlated with the expression of OPN and BMP2 target genes, which are typically positive regulators of osteogenesis (Figure S4D). These results suggest that EXO‐shSlo1 negatively regulates osteogenic transcription in osteoblasts.

### 3.5 MiR‐222‐3p is Abundant in Exosomes From Slo1‐Deficient Myoblasts

**FIGURE 5:**
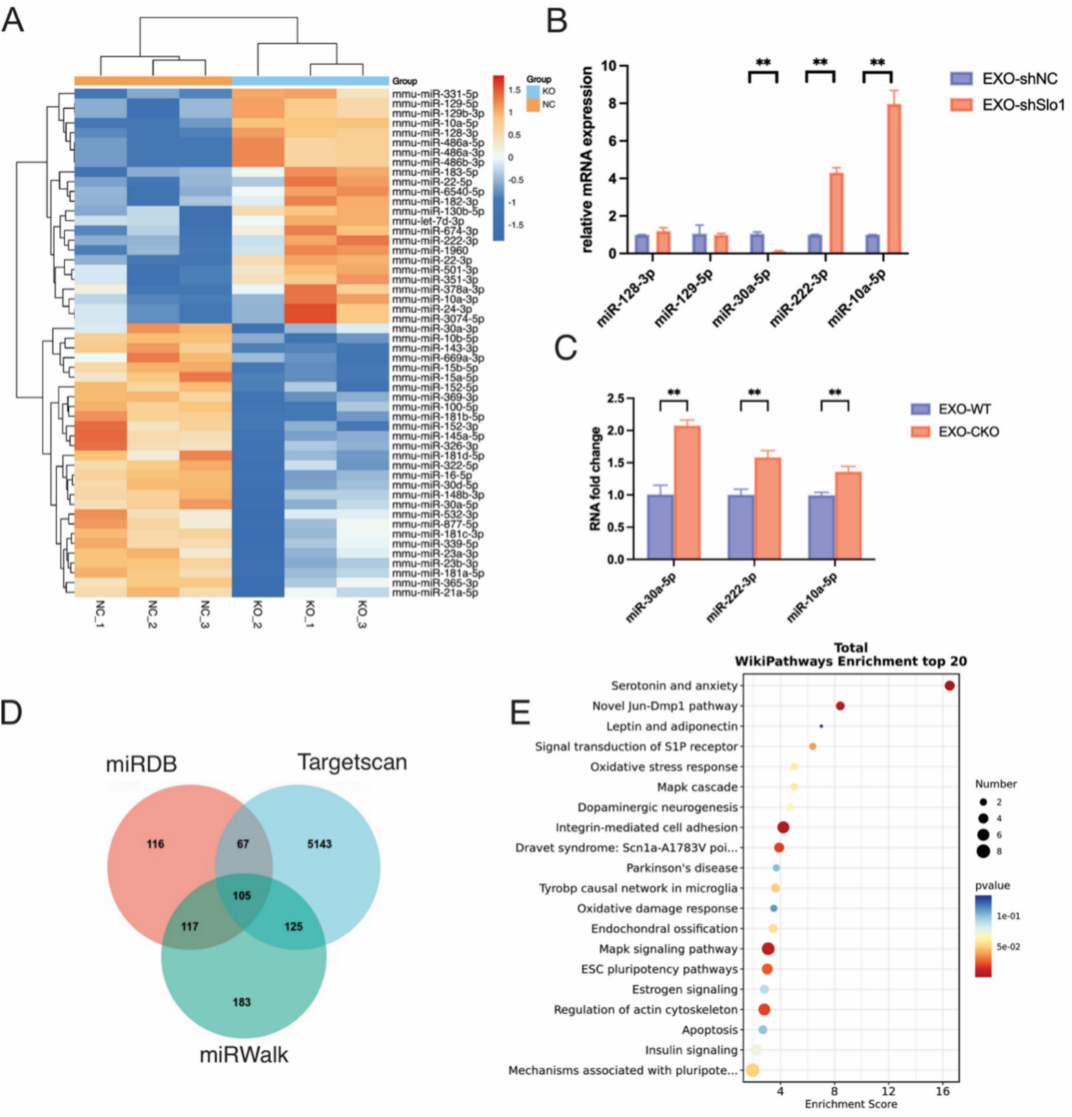
Exosomal miR‐222‐3p expression is increased when Slo1 is knocked down in myoblasts. (A) miRNAs expressed in the EXO‐shNC and EXO‐shSlo1 groups. The values represent the log2‐fold change in miRNA expression in the EXO‐shSlo1 group compared with to the control EXO‐shNC group. Blue and red indicate downregulation and upregulation, respectively. (B) RNA was extracted from EXO‐shNC and EXO‐shSlo1, and the relative expression levels of miR‐128‐3p, miR‐129‐5p, miR‐30‐5p, miR‐222‐3p and miR‐10a‐5p were measured by q‐PCR. (C) RNA was extracted from EXOs‐WT and EXOs‐CKO, and the relative expression levels of miR‐30‐5p, miR‐222‐3p and miR‐0a‐5p were measured by q–PCR. Representative osteogenesis related genes identified by GO enrichment. (D) The Venn diagram of predicted target genes of miR‐222‐3p in miRWalk, miRDB and Targetscan. (E) KEGG pathway analysis of overlapped target genes of miR‐222‐3p. *n* = 3; *p < 0.05, **p < 0.01 and ***p < 0.001. (EXO‐WT means the exosomes derived from myoblasts of WT mice; EXO‐CKO means the exosomes extracted from myoblasts of muscle‐specific Slo1 CKO mice).

We next investigated how Slo1‐deficient myoblast‐derived exosomes inhibit osteogenesis. MiRNAs encapsulated in exosomes are abundant and play a crucial role in cell–cell communication. To identify candidate miRNAs in EXO‐shSlo1 involved in mediating muscle‐bone communication, we examined the miRNA profiles of EXO‐shSlo1 and EXO‐shNC using small RNA sequencing. After normalization and filtering out miRNAs with fold changes > 1.5 or <−1.5, and *p*‐values < 0.05, we identified differentially expressed miRNAs. Specifically, 28 downregulated and 24 upregulated miRNAs were found in the EXO‐shSlo1 group (Figure 5A, Table S2).

We first assessed the ALP activity in MC3T3‐E1 cells of the top‐ranked miRNAs from the sequencing results. However, ALP staining and activity indicated that these 10 miRNAs were not associated with osteogenesis, as ALP activity in MC3T3‐E1 osteoblasts cells remained unchanged (Figure S5A–D). Next, we focused on miRNAs related to osteogenesis that matched our phenotype. Among these, miR‐128‐3p and miR‐129‐5p did not show differential expression between EXO‐shSlo1 and EXO‐shNC. As shown in Figure 5B, while the expression levels of miR‐30a‐5p and miR‐10a‐5p in EXO‐shSlo1 aligned with sequencing results, miR‐30a‐5p was expressed at higher levels in EXO‐CKO compared to EXO‐WT (Figure 5C). ALP staining in MC3T3‐E1 cells further revealed that miR‐30a‐5p and miR‐10a‐5p did not significantly affect ALP activity (Figure S5E,F).

In contrast, miR‐222‐3p exhibited the most significant changes. This miRNA has been reported to inhibit osteoblast differentiation [31, 32]. qPCR confirmed the presence of miR‐222‐3p in EXO‐shSlo1 and EXO‐CKO, showing elevated levels in EXO‐shSlo1 (Figure 5B,C). Therefore, miR‐222‐3p may be a key miRNA delivered to bones to inhibit osteogenesis. Finally, we analysed the predicted functions of miR‐222‐3p using miRDB, miRWalk and TargetScan to identify its target genes (Figure 5D). Enrichment analysis of the 105 overlapping targets revealed that miR‐222‐3p is associated with “endochondral ossification” and “MAPK signalling” (Figure 5E).

The ovariectomized (OVX) mice model was utilized to evaluate the association of miR‐222‐3p and bone mass. It shows that the mRNA expression levels of Col1a1 and ALP were decreased in bone from an OVX model (Figure S6A), and the miR‐222‐3p levels in bones, muscle and serum exosomes were greater in the OVX mice than in the sham mice (Figure S6B). Simultaneously, the RNA levels of miR‐222‐3p were observed to increase with advancing age in WT mice (Figure S6C). We examined the miR‐222‐3p in our CKO mice. As indicated in Figure S6D, miR‐222‐3p level was increased in the muscle, bones and blood of CKO mice compared to WT mice. These findings indicated that miR‐222‐3p is associated with bone loss.

### 3.6 miR‐222‐3p Inhibit Osteoblast Differentiation

**FIGURE 6:**
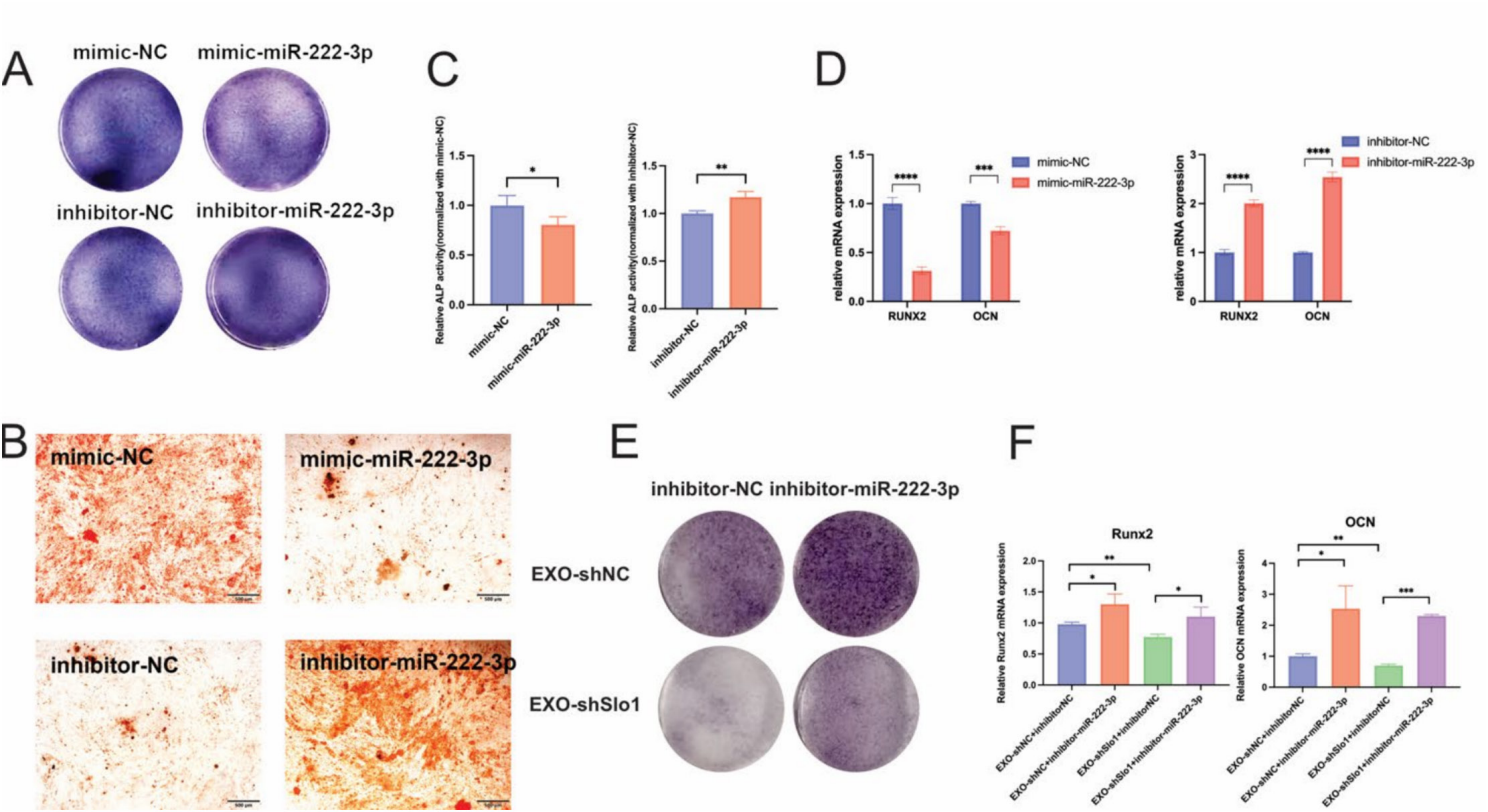
Exosomal miR‐222‐3p inhibits the osteogenic differentiation of MC3T3‐E1 cells. (A) MC3T3‐E1 cells were transfected with a NC mimic, miR‐222‐3p mimic, NC inhibitor or miR‐222‐3p inhibitor. The transfected cells were osteogenically induced for 7 days. Representative image of ALP staining. (B) Representative images of ARS staining after 21 days of osteogenic induction. (C) ALP activity was assessed after 7 days of osteogenic induction. (D) The relative mRNA expression of the Runx2 and OCN osteogenic markers in the NC mimic vs. miR‐222‐3p mimic (left panel) and NC inhibitor vs. miR‐222‐3p inhibitor (right panel) groups determined by q‐PCR. (E) MC3T3‐E1 cells were transfected with a NC inhibitor or miR‐222‐3p inhibitor, followed by treatment with EXO‐shNC and EXO‐shSlo1. The cells were then subjected to 7 days of osteogenic differentiation. Representative images of ALP staining. (F) RNA was extracted from MC3T3‐E1 cells, and the mRNA expression levels of Runx2 and OCN, and were measured by q–PCR. *n* = 3; *p < 0.05, **p < 0.01 and ***p < 0.001. (EXO‐shNC means the exosomes derived from C2C12 transfected with shNC; EXO‐shSlo1 means the exosomes extracted from C2C12 transfected with shSlo1).

For validation of the osteogenic effect of miR‐222‐3p on osteoblasts, MC3T3‐E1 cells were transfected with a mimic or inhibitor of miR‐222‐3p. ALP activity/staining and ARS staining demonstrated that the miR‐222‐3p mimic inhibited ALP levels and mineralization, while the miR‐222‐3p inhibitor increased ALP levels and mineralization (Figure 6A–C, S7A). qPCR was performed to evaluate the mRNA expression levels of the osteogenic markers Runx2 and OCN (Figure 6D). Consistent with previous results, the miR‐222‐3p mimic decreased the transcription of these osteogenic markers, while the miR‐222‐3p inhibitor significantly promoted the expression of these osteogenic markers. Additionally, the protein levels of the osteogenic marker Runx2 were influenced accordingly (Figure S7C). To further investigate the role of miR‐222‐3p in osteoporosis, we transfected miR‐222‐3p inhibitors into MC3T3‐E1 osteoblasts treated with EXO‐shSlo1. As illustrated in Figures 6E, S7B and S7D, the introduction of the miR‐222‐3p inhibitor significantly counteracted the reduction in ALP activity caused by EXO‐shSlo1. Additionally, qPCR analysis showed that the mRNA levels of osteogenic markers Runx2 and OCN increased following transfection with the inhibitors (Figure 6F). Western blotting further assessed the protein levels of these osteogenic markers and indicated that Runx2, ALP and OPN were more highly expressed in the group receiving both EXO‐shSlo1 and the miR‐222‐3p inhibitor (Figure S7E). These findings suggest that inhibition of miR‐222‐3p alleviates the suppression of osteoblasts. Therefore, these findings demonstrated that miR‐222‐3p is a critical component that regulates the differentiation of osteoblasts.

### 3.7 miR‐222‐3p in EXO‐shSlo1 Plays a Critical Role in Detrimental Skeletal Effects by Targeting STAT3

**FIGURE 7:**
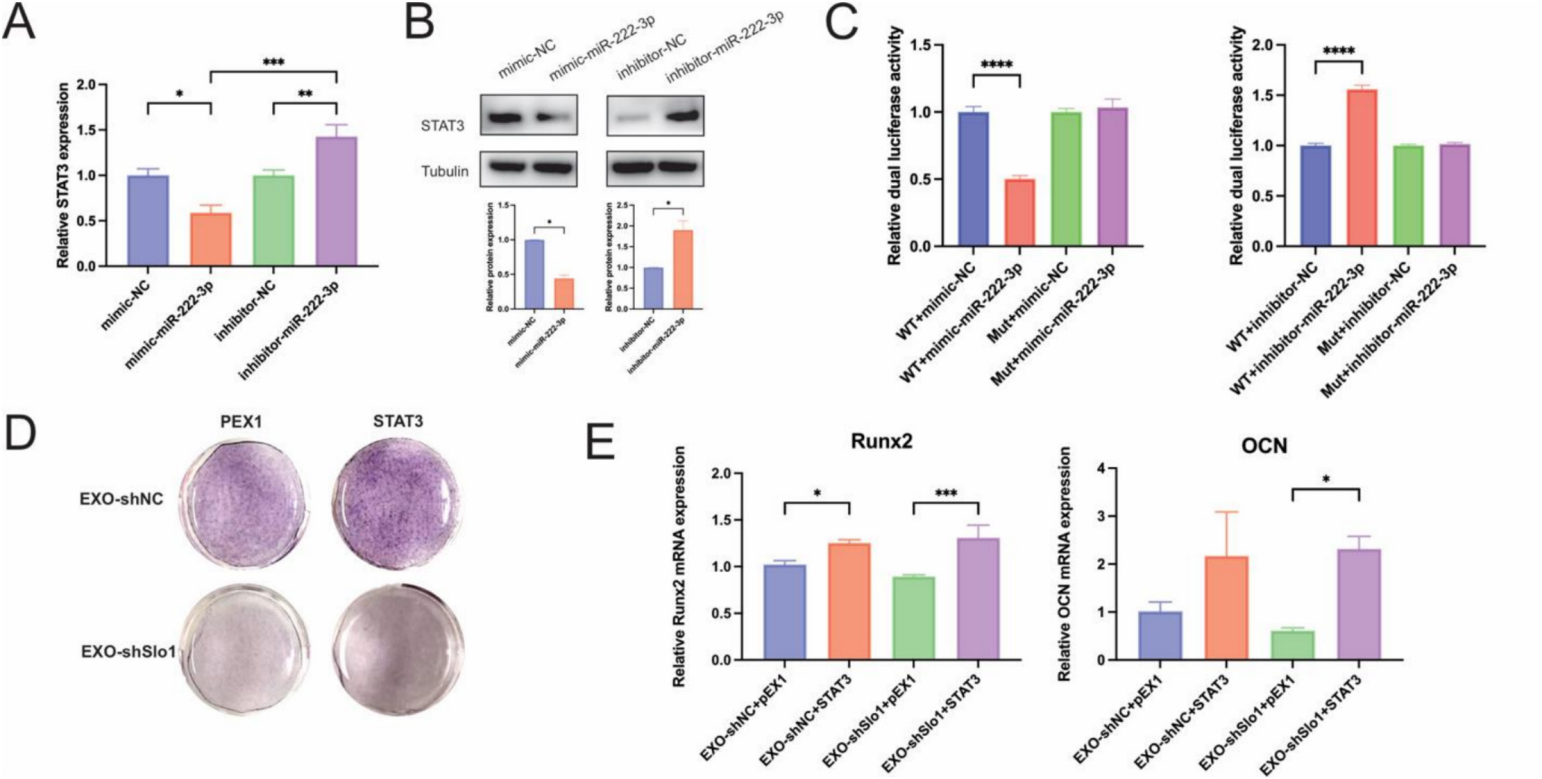
miR‐222‐3p targets STAT3 to regulate osteogenesis. (A) MC3T3‐E1 cells were transfected with NC mimic, miR‐222‐3p mimic, NC inhibitor or miR‐222‐3p inhibitor. The mRNA expression level of STAT3 was analysed by q‐PCR. (B) The protein level of STAT3 was analysed by WB. (C) Luciferase activity in the indicated MC3T3‐E1 cells after transfection of the miR‐222‐3p mimic, miR‐2223p inhibitor, NC mimic or NC inhibitor with the binding site mutant (Mut) or wild‐type 3′‐UTR‐driven reporter construct. (D) MC3T3‐E1 cells were transfected with a vector control, PEX1 or STAT3, followed treatment with EXO‐shNC or EXO‐shSlo1. The cells were then subjected to 7 days of osteogenic differentiation. Representative images of ALP staining. (E) RNA was extracted from MC3T3‐E1 cells, and the mRNA expression levels of Runx2 and OCN were measured by q–PCR. *n* = 3, *p < 0.05, **p < 0.01 and ***p < 0.001.

To investigate the mechanism by which miR‐222‐3p mediates the osteogenic inhibition of EXO‐shSlo1, we examined the mRNA expression levels of several osteogenesis‐related predicted targets of miR‐222‐3p, including LRP5, LRP6, ATF4, CBFB, STAT3 and IGF‐1. As illustrated in Figure 7A and S8A, transfection MC3T3‐E1 cells with miR‐222‐3p significantly altered the mRNA expression level of STAT3, while the expression levels of other targets remained unchanged. Moreover, both the miR‐222‐3p mimic and inhibitor affected the protein level of STAT3 (Figure 7B), indicating that miR‐222‐3p interacts with STAT3 and affect its transcription. Additionally, the luciferase reporter assay demonstrated that transfection with the miR‐222‐3p mimic and the 3′UTR of STAT3 significantly reduced luciferase activity, whereas the mutant 3′UTR of STAT3 and the negative control did not affect luciferase activity (Figure 7C). Furthermore, the miR‐222‐3p inhibitor combined with the wild‐type 3′UTR of STAT3 notably increased luciferase activity, while the miR‐222‐3p inhibitor combined with the mutant 3′UTR of STAT3 did not produce similar effects. Schematic diagram of mutant STAT3, wild‐type STAT3 and miR‐222‐3p binding sites as shown in Figure S8B. These results suggest that miR‐222‐3p binds to the 3′UTR of STAT3, thereby influencing its transcription. To further explore the role of STAT3, we overexpressed STAT3 in MC3T3‐E1osteoblasts (Figure S8C,D). ALP staining and activity assays were conducted after transfection with STAT3 or an empty vector into EXO‐shNC‐ or EXO‐shSlo1‐treated MC3T3‐E1 cells (Figure 7D, S8E,F). STAT3 overexpression enhanced ALP activity in both EXO‐shNC‐ and EXO‐shSlo1‐treated MC3T3‐E1 cells. Furthermore, qPCR analysis revealed that STAT3 overexpression mitigated the EXO‐shSlo1‐induced suppression of osteogenic marker transcription (Figure 7E). Additionally, protein levels of Runx2, ALP and OPN were restored in the EXO‐shSlo1 group following STAT3 overexpression (Figure S8G). These results indicate that miR‐222‐3p critically influences the EXO‐shSlo1‐mediated osteogenic inhibition effect by targeting STAT3.

## 4 Discussion

Osteosarcopenia is characterized by the concurrent presence of sarcopenia and osteoporosis. The mechanism of Osteosarcopenia remains unclear. There are several osteosarcopenia models to be studied. Aging is a significant risk factor for musculoskeletal disorders, with 18‐month‐old rats frequently utilized to establish natural aging osteoporosis (OP) models, whereas high‐fat diets and senescence‐accelerated prone mice (SAMP) are employed to expedite the aging process [32, 33, 34]. The ovariectomy (OVX) model, which involves the surgical removal of rat ovaries, results in a decline in oestrogen levels, leading to the development of osteoporosis and skeletal muscle atrophy, thereby effectively replicating OS pathology [33]. Additionally, chemical induction and disuse models are used to induce OP through the administration of glucocorticoids or the reduction of skeletal loading, respectively, while genetic models are employed to simulate chronic inflammation and osteoporosis through gene knockout or mutation techniques [35, 36].

In this study, we found that Slo1 CKO mice exhibit decreased BMD, further supporting the evidence for bone‐muscle crosstalk. Mechanical stimulation clearly plays a critical role in bone loss in Slo1 CKO mice. This study shifts focus to other factors involved in bone‐muscle crosstalk in these mice.

It is acknowledged that secreted factors and exosomes mutually influence bone and muscle. Extracellular vesicles are important transporters that deliver signals between cells. According to the current knowledge of biogenesis, extracellular vesicles can be divided into two categories, namely, exosomes and microvesicles [37]. Specifically, exosomes have a diameter of 30 ~ 100 nm, and they are intraluminal vesicles that are formed by inward budding of the endosomal membrane. Exosomes are secreted by the fusion of multivesicular endosomes with the cell surface [38]. In contrast, microvesicles are larger, measuring 50–1000 nm in diameter, and are produced by outward budding and fission of the plasma membrane before being released into the extracellular space [39]. Extracellular vesicles are now recognized as key players in bone‐muscle crosstalk. For example, BMSC‐derived exosomes alleviate muscle atrophy by delivering miR‐486‐5p to muscle cells [40]. They also improve muscle atrophy and healing by regulating macrophage polarization [S1]. Under healthy conditions, they promote bone formation and repair; however, during aging or in disease states (such as muscle atrophy or chronic kidney disease), dysregulation of exosomal miRNAs contributes to bone loss and muscle wasting [S2]. In vitro, C2C12 myoblast‐derived exosomes enhance MC3T3‐E1 osteogenic differentiation through miR‐27a‐3p‐dependent APC suppression, which potentiates β‐catenin signalling to drive osteogenesis [S3]. On the contrary, it was demonstrated that exosomes derived from atrophic muscles suppress osteogenic differentiation of BMSCs while enhancing osteoclast activity [S4]. Senescent muscle can result from exosomal miR‐34a derived from senescent BMSCs [S5], and mechanical stimulation of muscle has been shown to improve bone mineral density (BMD) by delivering exosomal miR‐92a‐3p [S6]. Our findings indicate that muscle‐derived exosomes localize in bone and osteoblasts, aligning with previous studies. Nowadays, the mechanism of exosome targeting cells remains understudied. We supposed that some proteins in C2C12‐derived exosomes contribute to the skeletal targeting. As reported exosomes might expressed integrins and CD47, which facilitate their interaction with hepatic and osseous tissues [S7, S8]. Notably, integrins play a pivotal role in mediating these biological processes [S9]. The bone‐targeting proteins in C2C12‐derived exosomes requires further tests to identify. Furthermore, several studies have demonstrated the potential of exosomes to deliver therapeutic components, suggesting they may serve as both a target for and a tool in future therapies. In premilitary assay, we screened the osteogenic effects of exosomes from 2.5 to 50 μg/mL in MC3G3‐E1 cells by ALP staining/activity and assessed the viability of exosomes by CCK8 (data not shown). Finally, the optimal concentration was determined by obvious osteogenic inhibition with little cytotoxicity (2.5 μg/mL). Then, we focused on exosome‐mediated bone‐muscle crosstalk in Slo1 CKO mice. RNA‐seq analysis comparing MC3T3‐E1 cells treated with EXO‐shSlo1 and EXO‐shNC revealed altered cellular transcription, with GO analysis indicating that EXO‐shSlo1‐treated osteoblasts were associated with osteoblast development. Initial analysis suggested that EXO‐shSlo1 modified the transcriptional profile and physiology of osteoblasts. To further investigate whether EXO‐shSlo1 interferes with osteoblast differentiation, we conducted ALP staining, ALP activity assays, qPCR, Western blotting (WB), and ARS staining to assess osteogenic maturation. The data demonstrated that EXO‐shSlo1 delayed osteogenic differentiation in vitro. In vivo, EXO‐shSlo1 induction affected trabecular microstructure and BMD when injected into the tail vein. Thus, EXO‐shSlo1 contributes, in part, to bone loss in Slo1 CKO mice.

Various exosomal miRNAs were delivered to target cells, influencing their function. RNA‐seq analysis of exosomal miRNA levels revealed 52 differentially expressed miRNAs in EXO‐shSlo1, which are implicated in osteogenic inhibition. Among the 10 most significantly expressed miRNAs identified through sequencing, none were found to influence ALP activity, as confirmed by ALP staining (Figure S5A–D). This may be because muscle‐derived exosomes can transport signals to other organs, suggesting that these top‐ranked miRNAs might exert their effects elsewhere. Future studies could uncover additional roles of muscle‐derived exosomes and the non‐osteogenic effects of exosomal miRNAs. We further analysed osteogenesis‐related miRNAs using qPCR and ALP staining. Notably, miR‐222‐3p was significantly altered in both EXO‐shSlo1 and EXO‐CKO, and it was found to suppress osteoblastic differentiation. Enrichment analysis revealed that miR‐222‐3p targets osteogenic genes. In the OVX model, high miR‐222‐3p expression was associated with reduced bone mass. Moreover, ALP staining, qPCR and ARS staining confirmed that miR‐222‐3p inhibits osteoblastic differentiation. Overall, consistent with previous research, miR‐222‐3p inhibits osteoblast differentiation.

Zhou et al. reported that miR‐222‐3p suppresses osteoblastic differentiation in hBMSCs [31], and another study revealed that downregulated miR‐222‐3p protects BMSCs from high glucose [32]. Similarly, miR‐222‐3p has been associated with osteogenesis and shown to inhibit osteogenesis in our study. Our findings indicated that transfection with miR‐222‐3p mimics decreased ALP activity, osteogenic marker expression and mineralization, whereas treatment with a miR‐222‐3p inhibitor increased ALP activity, osteogenic marker expression and mineralization. Overall, exosomal miR‐222‐3p inhibits osteogenesis.

Furthermore, IGF‐1 is a known target of miR‐222‐3p in inhibiting osteogenic differentiation [32]. However, in our study, qPCR revealed no significant change in the mRNA expression level of IGF‐1 following miR‐222‐3p transfection. A possible explanation is that miRNAs can target different genes to exert various functions. With integration of prediction of TargetScan, miRDB and miRWalk, we found that STAT3 is a potential target of miR‐222‐3p. A dual‐fluorescence reporter assay demonstrated that miR‐222‐3p binds to the 3′UTR of STAT3, significantly reducing both transcript and protein levels. Additionally, the expression levels of other predicted targets were not affected by miR‐222‐3p (Figure S7A). STAT3 is a downstream factor in the MAPK signalling pathway, a well‐established osteogenic transcription factor [S10, S11]. Thus, this study shows that exosome‐mediated communication from muscle to bone leads to bone loss, and miR‐222‐3p inhibitors may offer therapeutic potential for sarcopenia and osteoporosis.

In summary, the present findings demonstrate that exosomal miR‐222‐3p is a potential messenger from muscle to bone, inhibiting bone formation by targeting STAT3 transcription. While previous studies have used aging or hindlimb suspension models to investigate the associations between sarcopenia and osteoporosis, this study employed a novel transgenic sarcopenia model, offering additional insights into bone–muscle crosstalk. However, the study lacked direct therapeutic evidence to confirm that miR‐222‐3p inhibition improves bone mass of CKO mice. Additionally, the other non‐exosomal factors were not elucidated in this research. A second limitation of this study concerns the age of the experimental animals. Mechanistic investigations were primarily conducted in 6 ~ 8‐week‐old mice to characterize the early phenotype of osteosarcopenia, thereby providing critical insights into potential targets for intervention during the preclinical stage of the disorder. Other researchers' experiments in adult mice were performed to evaluate the role of the oestrogen receptor in regulating muscle mass through assessment of contractile properties [S12]. Nonetheless, because osteosarcopenia is an age‐associated condition that predominantly affects elderly individuals, the present findings do not fully reflect the pathophysiological manifestations of sarcopenic osteoporosis in the context of aging. Future work will be required to elucidate the specific contributions of aging to the development of osteosarcopenia. A further limitation lies in the use of sex‐mixed cohorts for CKO phenotype assessment. Given that sarcopenia is strongly modulated by sex hormones, with males generally exhibiting greater susceptibility to muscle loss, combining sexes in the analysis may introduce biological variability. However, inclusion of both sexes also enhances statistical power and provides a more comprehensive understanding of the condition, consistent with prior studies that have examined sarcopenia in mixed‐sex cohorts [S13‐S16]. That said, sex‐stratified analyses remain essential for disentangling the influence of gender‐specific factors and minimizing potential confounders. Indeed, our preliminary experiments conducted separately in male and female CKO mice demonstrated bone loss in both sexes, underscoring the relevance of future sex‐specific investigations. The Future research aims to design modified exosomes targeting osteoblasts without affecting other cell functions and to package miR‐222‐3p inhibitors for more efficient osteogenesis and explore the effect of muscle‐secreting factors and mechanical stimuli on bones. In translational medicine, inhibiting miR‐222‐3p in osteoblasts may serve as a potential bone anabolic strategy to alleviate sarcopenia‐related osteoporosis.

## Funding

The present study was sponsored by the National Key R&D Program of China (2023YFC3605705), the Fundamental Research Funds for the Central Universities (YG2024ZD09), the National Natural Science Foundation of China (82571807), the Science and Technology Commission of Shanghai Municipality (20ZR1435100), the Shanghai Municipal Health Commission (2020YJZX0122).

## Conflicts of Interest

The authors declare no conflicts of interest.

## Supporting information

Table 1. Primers for RT‐PCR.
Table 2. RNAseq results.

**Data S1:** Supplementary Information.

**Figure S1:** Slo1 CKO does not influence cortical bone. (A) The protein level of Slo1 in WT and CKO bones. (B) The mRNA level of Slo1 in WT and CKO bones. (C) Cortical bone of 3D reconstructed tibias from WT and CKO mice. (D) Quantitative analysis of the bone area per total area (BA/TA) and cortical thickness (Ct. Th) of WT and CKO tibias (*n* = 7), (E) Sex‐based analysis of micro‐CT of CKO and WT mice (4 male, 3 female mice)*p < 0.05, **p < 0.01 and ***p < 0.001.
**Figure S2:** The mRNA expression level of Slo1 in C2C12 cells after infection with Ad‐NC, Ad‐shSlo1‐1, AdshSlo1‐2, or Ad‐shSlo1‐3. n = 3, *p < 0.05, **p < 0.01 and ***p < 0.001.
**Figure S3:** EXO‐WT inhibits the osteogenic differentiation of MC3T3‐E1 cells. (A) MC3T3‐E1 cells were treated with EXO‐WT or EXO‐CKO and induced with osteogenic differentiation medium for 7 days. Representative images of ALP staining. (B) ALP activity was quantified after 7 days of induction with PBS, EXO‐WT or EXO‐CKO. (C) The protein levels of ALP, OPN and Runx2 were evaluated by WB. (D) mRNA expression levels of Runx2 and ALP in PBS‐, EXO‐WT‐ and EXO‐CKO‐treated MC3T3‐E1 cells. n = 3; *p < 0.05, **p < 0.01 and ***p < 0.001.
**Figure S4:** RNA‐seq analysis revealed that the differentially expressed mRNAs are associated with osteogenesis. (A) Heatmap of hierarchical clusters of differentially expressed mRNAs in MC3T3‐E1 cells induced with EXO‐shNC or EXO‐shSlo1. The values represent the log2‐fold change in the mRNA levels of EXO‐shSlo1‐treated MC3T3‐E1 cells compared with those of the control EXO‐shNC‐treated MC3T3‐E1 cells. Blue and red indicate downregulation and upregulation, respectively. (B) GO analysis of differentially expressed mRNAs. (C) KEGG pathway analysis of the differentially expressed mRNAs. (D) GSEA analysis.
**Figure S5:** MC3T3‐E1 cells were transfected with miR‐369‐3p, miR‐181c‐3p, miR‐326‐3p, miR‐15a‐5p, miR‐15b‐5p, which are top 5 low expressed miRNAs in miRNAs sequencing, and induced with osteogenic differentiation medium for 7 days: (A) Representative images of ALP staining; (B) The ALP activity after transfection of top 5 miRNAs in MC3T3‐E1 cell. MC3T3‐E1 cells were transfected with miR‐130b‐5p, miR‐486a‐5p, miR‐486b‐3p, miR‐486a‐3p, miR‐182‐3p, which are top 5 high expressed miRNAs in miRNAs sequencing, and induced with osteogenic differentiation medium for 7 days: (C) Representative images of ALP staining; (D) The ALP activity after transfection of top 5 miRNAs in MC3T3‐E1 cells. MC3T3‐E1 cells were transfected with miR‐10a‐5p, miR‐30a‐5p, which are reported osteogenesis‐related miRNAs, and induced with osteogenic differentiation medium for 7 days: (E) Representative images of ALP staining; (F) The ALP activity after transfection of osteogenic miRNAs in MC3T3‐E1 cells. n = 3, *p < 0.05, **p < 0.01 and ***p < 0.001.
**Figure S6:** (A) RNA was extracted from the bones of sham and OVX mice, and the relative mRNA expression levels of ALP and Col1a1 were measured by q‐PCR. (B) The relative expression level of miR‐222‐3p in the muscle, bones and blood of sham and OVX mice was measured by q‐PCR. (C) RNA was extracted from muscles from 3‐month, 12‐month and 24‐month‐old mice, and the relative expression level of miR‐222‐3p was assessed by q‐PCR. (D) The relative expression level of miR‐222‐3p in the muscle, bones and blood of WT and CKO mice was measured by q‐PCR. n = 3; *p < 0.05, **p < 0.01 and ***p < 0.001.
**Figure S7:** (A) The represented ALP staining images of MC3T3‐E1 cells transfected with mimic and inhibitor of miR‐222‐3p. MC3T3‐E1 cells were transfected with a NC inhibitor or miR‐222‐3p inhibitor, followed by treatment with EXO‐shNC and EXO‐shSlo1, the cells were then subjected to 7 days of osteogenic differentiation. (B) Representative images of ALP staining. (C) The protein levels of Runx2 were evaluated by WB. (D). The ALP activity of MC3T3‐E1 cells transfected with a NC inhibitor or miR‐222‐3p inhibitor, followed by treatment with EXO‐shNC and EXO‐shSlo1. (E) The protein levels of ALP, OPN, and Runx2 were measured by WB. n = 3, *p < 0.05, **p < 0.01 and ***p < 0.001. (EXO‐shNC means the exosomes derived from C2C12 transfected with shNC; EXO‐shSlo1 means the exosomes extracted from C2C12 transfected with shSlo1).
**Figure S8:** (A)Q‐PCR analysis of predicted target of miR‐222‐3p. (B) Schematic diagram of mutant STAT3, wild‐type STAT3, and miR‐222‐3p binding sites. (C) The mRNA expression level of STAT3 after transfection of PEX1 and STAT3. (D) STAT3 protein levels after transfection with PBS, PEX1, or STAT3. (E) MC3T3‐E1 cells were transfected with a vector control, PEX1 or STAT3, followed treatment with EXO‐shNC or EXO‐shSlo1. The cells were then subjected to 7 days of osteogenic differentiation. Representative images of ALP staining. (F) Quantitative ALP activity was measured. (G) The protein levels of ALP, OPN, and Runx2 were evaluated by WB. n = 3, *p < 0.05, **p < 0.01 and ***p < 0.001. (EXO‐shNC means the exosomes derived from C2C12 transfected with shNC; EXO‐shSlo1 means the exosomes extracted from C2C12 transfected with shSlo1).
