# Supplementary material for: Slo1 Deficient Myoblast Exosomes‐Derived miR‐222‐3p Inhibits Osteogenic Differentiation via Targeting of STAT3
Source: J Cachexia Sarcopenia Muscle. 2025 Dec 8;16(6):e70115. doi: 10.1002/jcsm.70115 (PMC12685407; doi:10.1002/jcsm.70115)
Supplement: Supplementary file 1 — Table 1. Primers for RT‐PCR. Table 2. RNAseq results. [file JCSM-16-e70115-s002.docx]

Table 1. Primers for RT-PCR

| Primer | Sequence (5’-3’) |
| --- | --- |
| Slo1-F | CGCCAGCCGTCCATCACAAC |
| Slo1-R | GTCAGGGTCATCGTCATCGTCTTG |
| Runx2-F | TGAGGGATGAAATGCTTGGGAACTG |
| Runx2-R | GATGATGACACTGCCACCTCTGAC |
| Col1a-F | GCTCCTCTTAGGGGCCACT |
| Col1a-R | CCACGTCTCACCATTGGGG |
| ALP-F | GACTGGTACTCGGATAACGA |
| ALP-R | TGCGGTTCCAGACATAGTGG |
| SP7-F | CAAAGAAGCCATACGCTGAC |
| SP7-R | GTCCATTGGTGCTTGAGAAG |
| OCN-F | CTGACCTCACAGATCCCAAGC |
| OCN-R | TGGTCTGATAGCTCGTCACAAG |
| OPN-F | CCAGGTTTCTGATGAACAGTATCC |
| OPN-R | CATCGAGGGACTCCTTAGACTCAC |
| STAT3-F | CAATACCATTGACCTGCCGAT |
| STAT3-R | GAGCGACTCAAACTGCCCT |
| GAPDH-F | \| AGGTCGGTGTGAACGGATTTG \| \| --- \| |
| GAPDH-R | TGTAGACCATGTAGTTGAGGTCA |

Table 2. RNAseq results

| sRNA | CKO_readcount | NC_readcount | log2FoldChange | pval | padj |
| --- | --- | --- | --- | --- | --- |
| mmu-miR-22-3p | 18220.0236 | 4906.0851 | 1.8316 | 2.81E-16 | 7.74E-14 |
| mmu-miR-15b-5p | 38.8475947 | 298.115829 | -2.6179 | 4.10E-11 | 5.64E-09 |
| mmu-miR-16-5p | 61.6538096 | 235.75148 | -1.8166 | 1.07E-09 | 9.83E-08 |
| mmu-miR-23b-3p | 279.979591 | 1022.19498 | -1.7605 | 1.85E-09 | 1.02E-07 |
| mmu-miR-369-3p | 23.6164379 | 104.900366 | -1.9937 | 1.56E-09 | 1.02E-07 |
| mmu-miR-15a-5p | 17.2396586 | 311.275181 | -3.1746 | 2.54E-09 | 1.17E-07 |
| mmu-miR-10a-5p | 1699.86174 | 446.254514 | 1.7985 | 1.63E-08 | 6.39E-07 |
| mmu-miR-145a-5p | 88.5806346 | 323.279851 | -1.7239 | 3.29E-07 | 1.06E-05 |
| mmu-miR-486a-5p | 2127.59553 | 350.006419 | 2.2376 | 3.45E-07 | 1.06E-05 |
| mmu-miR-486a-3p | 2124.98222 | 352.02244 | 2.2245 | 4.63E-07 | 1.16E-05 |
| mmu-miR-486b-3p | 2108.10805 | 347.732708 | 2.2288 | 4.53E-07 | 1.16E-05 |
| mmu-miR-181a-5p | 548.071231 | 1294.56136 | -1.1889 | 2.01E-06 | 4.61E-05 |
| mmu-miR-128-3p | 3386.66987 | 939.074371 | 1.6854 | 2.95E-06 | 6.25E-05 |
| mmu-miR-130b-5p | 18.9585325 | 1.72403864 | 2.4424 | 7.61E-06 | 0.0001495 |
| mmu-miR-152-3p | 938.273137 | 2325.24187 | -1.2392 | 1.46E-05 | 0.000267 |
| mmu-miR-129-5p | 2389.38713 | 552.824226 | 1.8066 | 4.87E-05 | 0.00070428 |
| mmu-miR-129b-3p | 2389.01577 | 552.559659 | 1.8069 | 4.86E-05 | 0.00070428 |
| mmu-miR-183-5p | 2279.53943 | 709.548861 | 1.522 | 4.58E-05 | 0.00070428 |
| mmu-miR-326-3p | 8.170723 | 54.5527692 | -2.1132 | 4.73E-05 | 0.00070428 |
| mmu-miR-22-5p | 3758.60993 | 2329.65772 | 0.67761 | 6.93E-05 | 0.00095347 |
| mmu-miR-6540-5p | 27.4947453 | 6.39617119 | 1.8061 | 7.55E-05 | 0.00098915 |
| mmu-miR-674-3p | 964.755458 | 344.623256 | 1.358 | 0.00011784 | 0.001473 |
| mmu-miR-181c-3p | 2.72566161 | 19.5723974 | -2.0714 | 0.00012338 | 0.0014752 |
| mmu-miR-100-5p | 3186.31583 | 5900.92476 | -0.8599 | 0.00015002 | 0.001719 |
| mmu-miR-222-3p | 804.18502 | 427.41133 | 0.87763 | 0.00019854 | 0.0021839 |
| mmu-miR-182-3p | 17.3930746 | 2.13890309 | 2.1066 | 0.00028469 | 0.0028809 |
| mmu-miR-23a-3p | 987.917016 | 2878.07303 | -1.3861 | 0.00028541 | 0.0028809 |
| mmu-miR-30d-5p | 3864.84928 | 7211.36311 | -0.86655 | 0.00029493 | 0.0028809 |
| mmu-miR-322-5p | 5.04855051 | 24.722935 | -1.7897 | 0.00030381 | 0.0028809 |
| mmu-miR-501-3p | 2488.17363 | 1389.959 | 0.81033 | 0.00049002 | 0.0044919 |
| mmu-miR-339-5p | 5.47647851 | 24.2596733 | -1.6741 | 0.00092476 | 0.0082035 |
| mmu-miR-10b-5p | 83.5873576 | 240.479485 | -1.3469 | 0.001135 | 0.0097538 |
| mmu-miR-1960 | 17.2727679 | 4.07633686 | 1.6414 | 0.0013644 | 0.01137 |
| mmu-miR-10a-3p | 52.2399698 | 20.2637545 | 1.2294 | 0.0015515 | 0.012549 |
| mmu-miR-143-3p | 7594.41395 | 16710.8945 | -1.0524 | 0.0016191 | 0.012722 |
| mmu-miR-365-3p | 18.5758414 | 85.5187453 | -1.6718 | 0.001777 | 0.013575 |
| mmu-miR-152-5p | 22.1306195 | 52.0723876 | -1.1266 | 0.0021248 | 0.015793 |
| mmu-miR-378a-3p | 25644.2585 | 12943.6618 | 0.92627 | 0.002229 | 0.016131 |
| mmu-miR-148b-3p | 147.137031 | 242.872763 | -0.69624 | 0.0026794 | 0.018081 |
| mmu-miR-181d-5p | 231.257222 | 398.061311 | -0.75192 | 0.0026722 | 0.018081 |
| mmu-miR-30a-3p | 71.5186472 | 274.766783 | -1.537 | 0.0026957 | 0.018081 |
| mmu-miR-351-3p | 75.4099795 | 38.3204607 | 0.90761 | 0.0030912 | 0.02024 |
| mmu-miR-532-3p | 5.20614013 | 37.7064583 | -1.7656 | 0.0036474 | 0.023327 |
| mmu-miR-30a-5p | 1016.085 | 1658.28775 | -0.68056 | 0.0044384 | 0.02774 |
| mmu-miR-331-5p | 80.0415415 | 33.1502711 | 1.119 | 0.0054026 | 0.033016 |
| mmu-miR-181b-5p | 361.815464 | 612.823537 | -0.7287 | 0.0057945 | 0.034328 |
| mmu-miR-24-3p | 53770.4246 | 37044.7663 | 0.52501 | 0.0059823 | 0.034328 |
| mmu-miR-3074-5p | 53768.9392 | 37043.947 | 0.525 | 0.0059919 | 0.034328 |
| mmu-miR-877-5p | 47.8376829 | 92.8723818 | -0.88777 | 0.0062914 | 0.035309 |
| mmu-miR-21a-5p | 27316.1397 | 56333.6496 | -0.95524 | 0.0068903 | 0.037897 |
| mmu-let-7d-3p | 1267.74703 | 674.96944 | 0.84491 | 0.0088092 | 0.04747 |
| mmu-miR-669a-3p | 8.58483124 | 30.9638569 | -1.4174 | 0.0089762 | 0.04747 |
