# Supplementary material for: Slo1 Deficient Myoblast Exosomes‐Derived miR‐222‐3p Inhibits Osteogenic Differentiation via Targeting of STAT3
Source: J Cachexia Sarcopenia Muscle. 2025 Dec 8;16(6):e70115. doi: 10.1002/jcsm.70115 (PMC12685407; doi:10.1002/jcsm.70115)
Supplement: Supplementary file 2 — Data S1: Supplementary Information. [file JCSM-16-e70115-s001.docx]

Supplementary references

S1. Luo Z, Lin J, Sun Y, Wang C, Chen J. Bone Marrow Stromal Cell-Derived Exosomes Promote Muscle Healing Following Contusion Through Macrophage Polarization. Stem Cells Dev. 2021;30:135-48.

S2. Kirk B, Lombardi G, Duque G. Bone and muscle crosstalk in ageing and disease. Nat Rev Endocrinol. 2025;21:375-90. doi:10.1038/s41574-025-01088-x

S3. Xu Q, Cui Y, Luan J, Zhou X, Li H, Han J. Exosomes from C2C12 myoblasts enhance osteogenic differentiation of MC3T3-E1 pre-osteoblasts by delivering miR-27a-3p. Biochem Biophys Res Commun. 2018;498:32-7. doi:10.1016/j.bbrc.2018.02.144

S4. Huang H, Ma S, Xing X, Su X, Xu X, Tang Q, et al. Muscle-derived extracellular vesicles improve disuse-induced osteoporosis by rebalancing bone formation and bone resorption. Acta Biomater. 2023;157:609-24. doi:10.1016/j.actbio.2022.12.019

S5. Fulzele S, Mendhe B, Khayrullin A, Johnson M, Kaiser H, Liu Y, et al. Muscle-derived miR-34a increases with age in circulating extracellular vesicles and induces senescence of bone marrow stem cells. Aging (Albany NY). 2019;11:1791-803.

S6. Xu N, Cui G, Zhao S, Li Y, Liu Q, Liu X, et al. Therapeutic Effects of Mechanical Stress-Induced C2C12-Derived Exosomes on Glucocorticoid-Induced Osteoporosis Through miR-92a-3p/PTEN/AKT Signaling Pathway. Int J Nanomedicine. 2023;18:7583-603.

S7. Hoshino A, Costa-Silva B, Shen TL, Rodrigues G, Hashimoto A, Tesic Mark M, et al. Tumour exosome integrins determine organotropic metastasis. Nature. 2015;527:329-35. doi:10.1038/nature15756

S8. Luan Y, Zhang Y, Li S, Gao C, Ying X, Zhao S, et al. CD47 is a tumor cell-derived exosomal signature and regulates tumor immune microenvironment and immunotherapy responses. Transl Oncol. 2025;53:102291. doi:10.1016/j.tranon.2025.102291

S9. Soe ZY, Park EJ, Shimaoka M. Integrin Regulation in Immunological and Cancerous Cells and Exosomes. Int J Mol Sci. 2021;22:doi:10.3390/ijms22042193

S10. Wang SY, Jiang JH, Liu SY, Zhang J, Gao X, Liu H, et al. Interleukin 6 promotes BMP9-induced osteoblastic differentiation through Stat3/mTORC1 in mouse embryonic fibroblasts. Aging (Albany NY). 2023;15:718-33. doi:10.18632/aging.204504

S11. Wu M, Chen G, Li YP. TGF-β and BMP signaling in osteoblast, skeletal development, and bone formation, homeostasis and disease. Bone Res. 2016;4:16009. doi:10.1038/boneres.2016.9

S12. Brown M, Ning J, Ferreira JA, Bogener JL, Lubahn DB. Estrogen receptor-alpha and -beta and aromatase knockout effects on lower limb muscle mass and contractile function in female mice. Am J Physiol Endocrinol Metab. 2009;296:E854-61. doi:10.1152/ajpendo.90696.2008

S13. Czyżowska A, Brown J, Xu H, Sataranatarajan K, Kinter M, Tyrell VJ, et al. Elevated phospholipid hydroperoxide glutathione peroxidase (GPX4) expression modulates oxylipin formation and inhibits age-related skeletal muscle atrophy and weakness. Redox Biol. 2023;64:102761. doi:10.1016/j.redox.2023.102761

S14. Ehara A, Taguchi D, Nakadate K, Ueda S. Attractin deficiency causes metabolic and morphological abnormalities in slow-twitch muscle. Cell Tissue Res. 2021;384:745-56. doi:10.1007/s00441-021-03423-w

S15. Sakuma S, Zhu EY, Raices M, Zhang P, Murad R, D'Angelo MA. Loss of Nup210 results in muscle repair delays and age-associated alterations in muscle integrity. Life Sci Alliance. 2022;5:doi:10.26508/lsa.202101216

S16. Wei B, Lu Y, Jin JP. Deficiency of slow skeletal muscle troponin T causes atrophy of type I slow fibres and decreases tolerance to fatigue. J Physiol. 2014;592:1367-80. doi:10.1113/jphysiol.2013.268177
