# Supplementary material for: Slo1 Deficient Myoblast Exosomes‐Derived miR‐222‐3p Inhibits Osteogenic Differentiation via Targeting of STAT3
Source: J Cachexia Sarcopenia Muscle. 2025 Dec 8;16(6):e70115. doi: 10.1002/jcsm.70115 (PMC12685407; doi:10.1002/jcsm.70115)
Supplement: Supplementary file 3 — Figure S1: Slo1 CKO does not influence cortical bone. (A) The protein level of Slo1 in WT and CKO bones. (B) The mRNA level of Slo1 in WT and CKO bones. (C) Cortical bone of 3D reconstructed tibias from WT and CKO mice. (D) Quantitative analysis of the bone area per total area (BA/TA) and cortical thickness (Ct. Th) of WT and CKO tibias (n = 7), (E) Sex‐based analysis of micro‐CT of CKO and WT mice (4 male, 3 female mice)*p < 0.05, **p < 0.01 and ***p < 0.001. Figure S2: The mRNA expression level of Slo1 in C2C12 cells after infection with Ad‐NC, Ad‐shSlo1‐1, AdshSlo1‐2, or Ad‐shSlo1‐3. n = 3, *p < 0.05, **p < 0.01 and ***p < 0.001. Figure S3: EXO‐WT inhibits the osteogenic differentiation of MC3T3‐E1 cells. (A) MC3T3‐E1 cells were treated with EXO‐WT or EXO‐CKO and induced with osteogenic differentiation medium for 7 days. Representative images of ALP staining. (B) ALP activity was quantified after 7 days of induction with PBS, EXO‐WT or EXO‐CKO. (C) The protein levels of ALP, OPN and Runx2 were evaluated by WB. (D) mRNA expression levels of Runx2 and ALP in PBS‐, EXO‐WT‐ and EXO‐CKO‐treated MC3T3‐E1 cells. n = 3; *p < 0.05, **p < 0.01 and ***p < 0.001. Figure S4: RNA‐seq analysis revealed that the differentially expressed mRNAs are associated with osteogenesis. (A) Heatmap of hierarchical clusters of differentially expressed mRNAs in MC3T3‐E1 cells induced with EXO‐shNC or EXO‐shSlo1. The values represent the log2‐fold change in the mRNA levels of EXO‐shSlo1‐treated MC3T3‐E1 cells compared with those of the control EXO‐shNC‐treated MC3T3‐E1 cells. Blue and red indicate downregulation and upregulation, respectively. (B) GO analysis of differentially expressed mRNAs. (C) KEGG pathway analysis of the differentially expressed mRNAs. (D) GSEA analysis. Figure S5: MC3T3‐E1 cells were transfected with miR‐369‐3p, miR‐181c‐3p, miR‐326‐3p, miR‐15a‐5p, miR‐15b‐5p, which are top 5 low expressed miRNAs in miRNAs sequencing, and induced with osteogenic differentiation medium f [file JCSM-16-e70115-s003.docx]

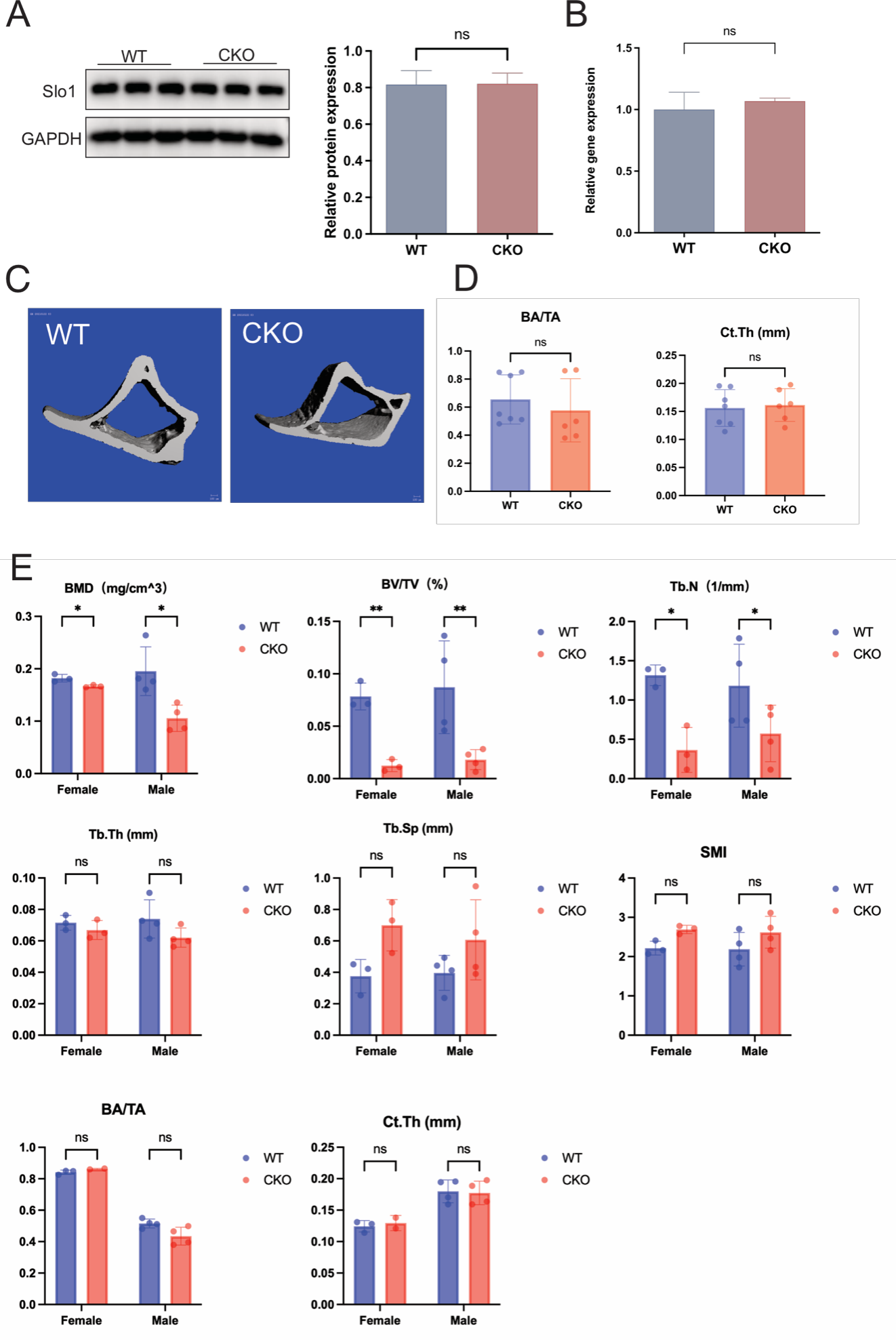


Supplementary 1. Slo1 CKO does not influence cortical bone. (A) The protein level of Slo1 in WT and CKO bones. (B) The mRNA level of Slo1 in WT and CKO bones. (C) Cortical bone of 3D reconstructed tibias from WT and CKO mice. (D) Quantitative analysis of the bone area per total area (BA/TA) and cortical thickness (Ct. Th) of WT and CKO tibias (n=7), (E) Sex-based analysis of micro-CT of CKO and WT mice (4 male, 3 female mice)*P<0.05, **P<0.01, and ***P<0.001.





Supplementary 2. The mRNA expression level of Slo1 in C2C12 cells after infection with Ad-NC, Ad-shSlo1-1, AdshSlo1-2, or Ad-shSlo1-3. n=3, *P<0.05, **P<0.01, and ***P<0.001.


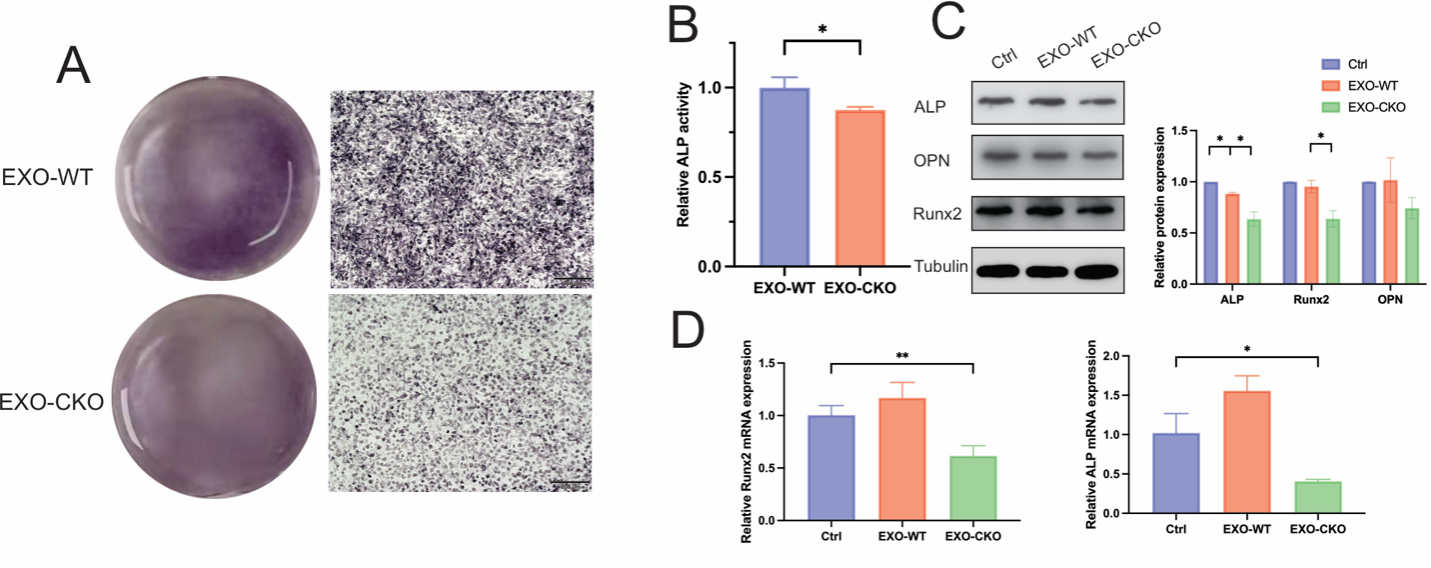
Supplementary 3. EXO-WT inhibits the osteogenic differentiation of MC3T3-E1 cells. (A) MC3T3-E1 cells were treated with EXO-WT or EXO-CKO and induced with osteogenic differentiation medium for 7 days. Representative images of ALP staining. (B) ALP activity was quantified after 7 days of induction with PBS, EXO-WT, or EXO-CKO. (C) The protein levels of ALP, OPN, and Runx2 were evaluated by WB. (D) mRNA expression levels of Runx2 and ALP in PBS-, EXO-WT-, and EXO-CKO-treated MC3T3-E1 cells. n=3; *P<0.05, **P<0.01, and ***P<0.001.


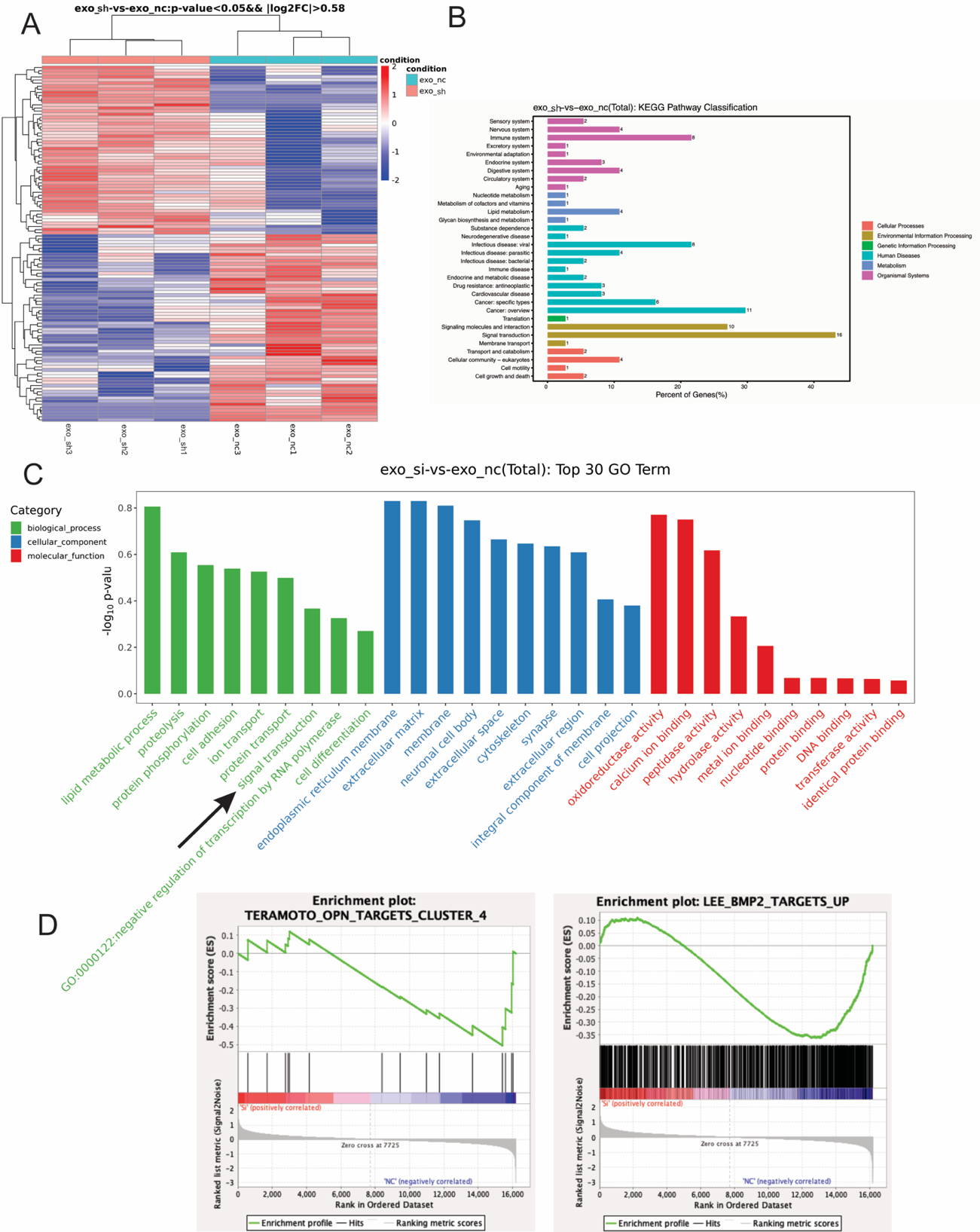
Supplementary 4. RNA-seq analysis revealed that the differentially expressed mRNAs are associated with osteogenesis. (A) Heatmap of hierarchical clusters of differentially expressed mRNAs in MC3T3-E1 cells induced with EXO-shNC or EXO-shSlo1. The values represent the log2-fold change in the mRNA levels of EXO-shSlo1-treated MC3T3-E1 cells compared with those of the control EXO-shNC-treated MC3T3-E1 cells. Blue and red indicate downregulation and upregulation, respectively. (B) GO analysis of differentially expressed mRNAs. (C) KEGG pathway analysis of the differentially expressed mRNAs. (D) GSEA analysis.


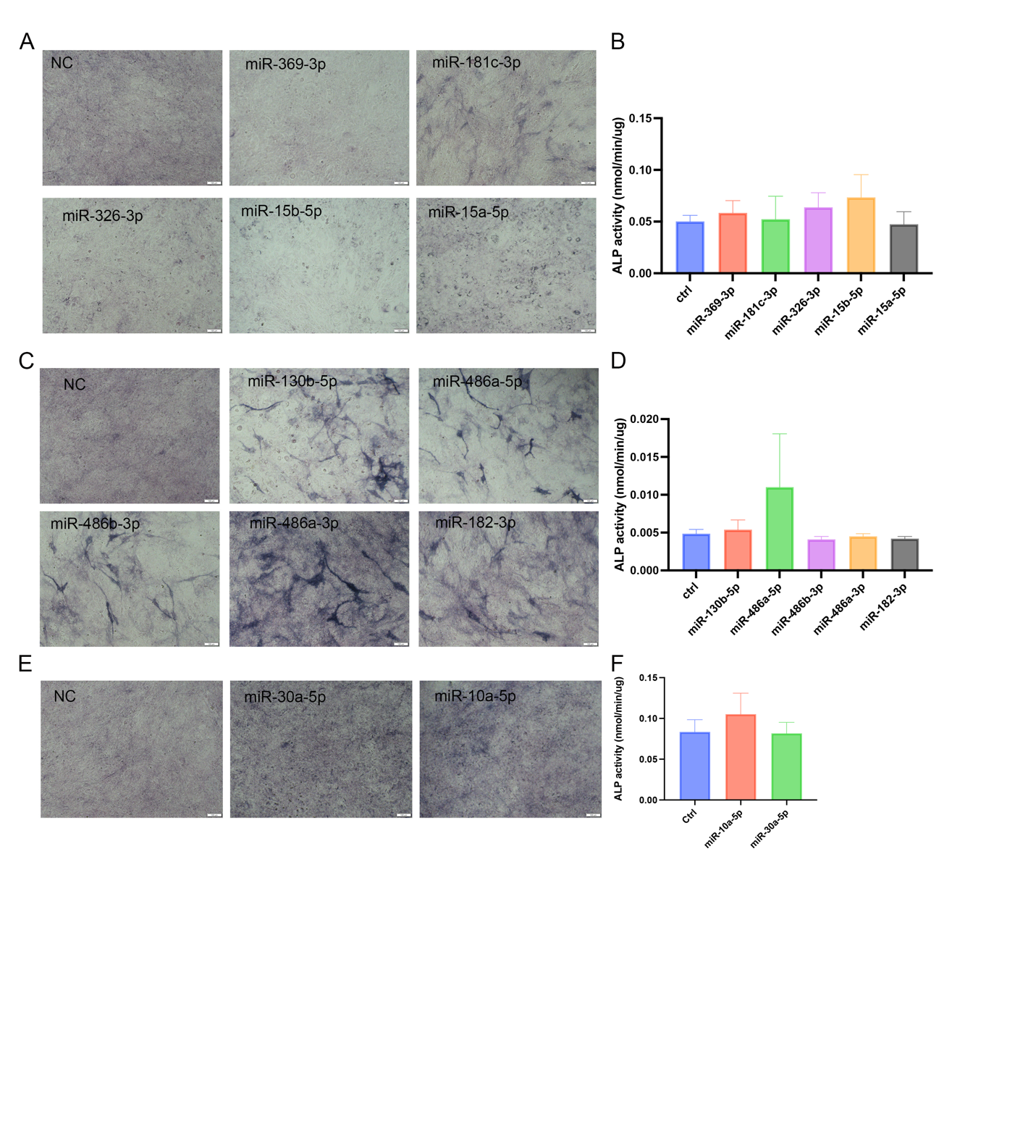


Supplementary 5. MC3T3-E1 cells were transfected with miR-369-3p, miR-181c-3p, miR-326-3p, miR-15a-5p, miR-15b-5p, which are top 5 low expressed miRNAs in miRNAs sequencing, and induced with osteogenic differentiation medium for 7 days: (A) Representative images of ALP staining; (B) The ALP activity after transfection of top 5 miRNAs in MC3T3-E1 cell. MC3T3-E1 cells were transfected with miR-130b-5p, miR-486a-5p, miR-486b-3p, miR-486a-3p, miR-182-3p, which are top 5 high expressed miRNAs in miRNAs sequencing, and induced with osteogenic differentiation medium for 7 days: (C) Representative images of ALP staining; (D) The ALP activity after transfection of top 5 miRNAs in MC3T3-E1 cells. MC3T3-E1 cells were transfected with miR-10a-5p, miR-30a-5p, which are reported osteogenesis-related miRNAs, and induced with osteogenic differentiation medium for 7 days: (E) Representative images of ALP staining; (F) The ALP activity after transfection of osteogenic miRNAs in MC3T3-E1 cells. n=3, *P<0.05, **P<0.01, and ***P<0.001.


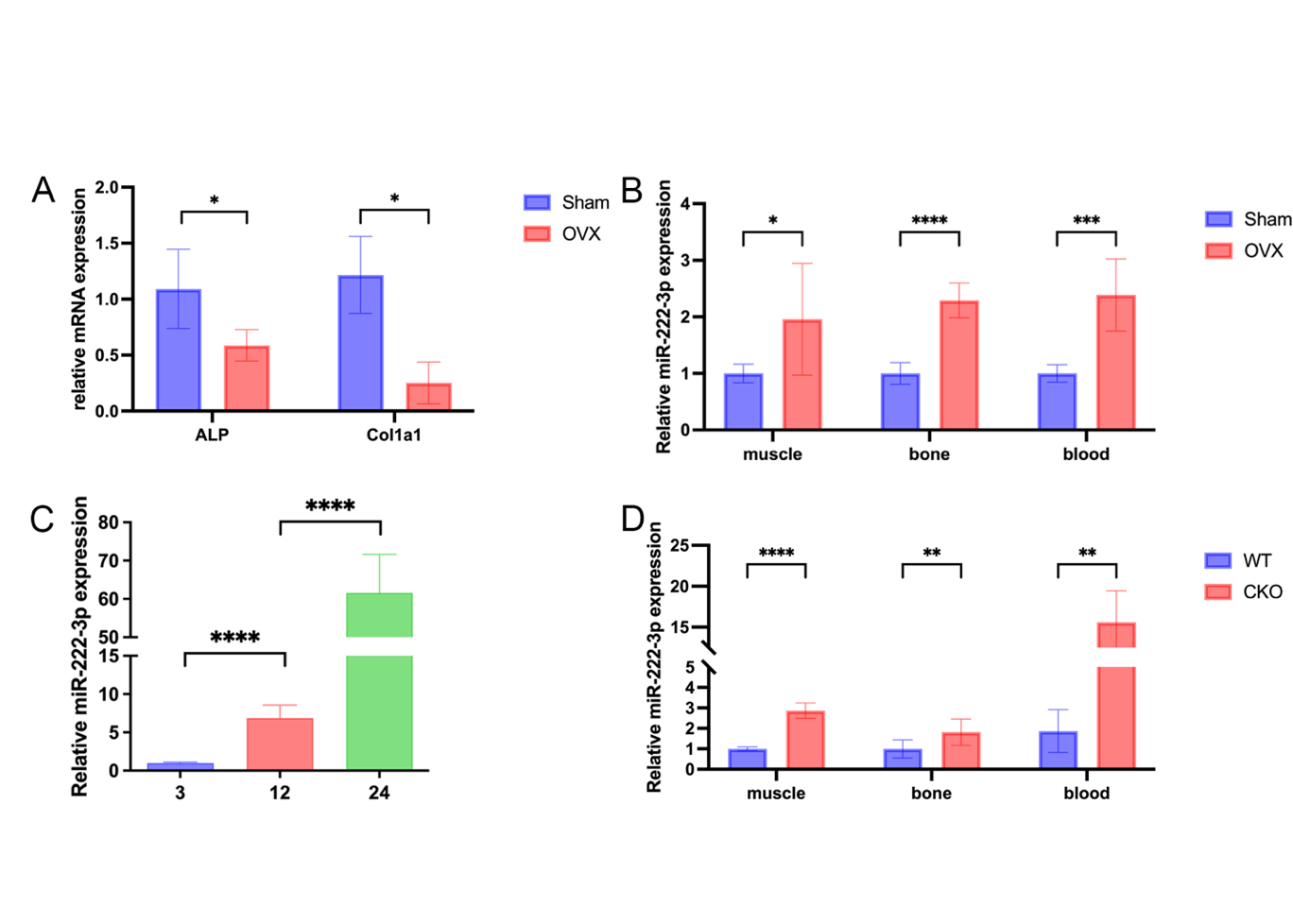


Supplementary 6. (A) RNA was extracted from the bones of sham and OVX mice, and the relative mRNA expression levels of ALP and Col1a1 were measured by q-PCR. (B) The relative expression level of miR-222-3p in the muscle, bones and blood of sham and OVX mice was measured by q-PCR. (C) RNA was extracted from muscles from 3-month, 12-month and 24-month-old mice, and the relative expression level of miR-222-3p was assessed by q-PCR. (D) The relative expression level of miR-222-3p in the muscle, bones and blood of WT and CKO mice was measured by q-PCR. n=3; *P<0.05, **P<0.01, and ***P<0.001.


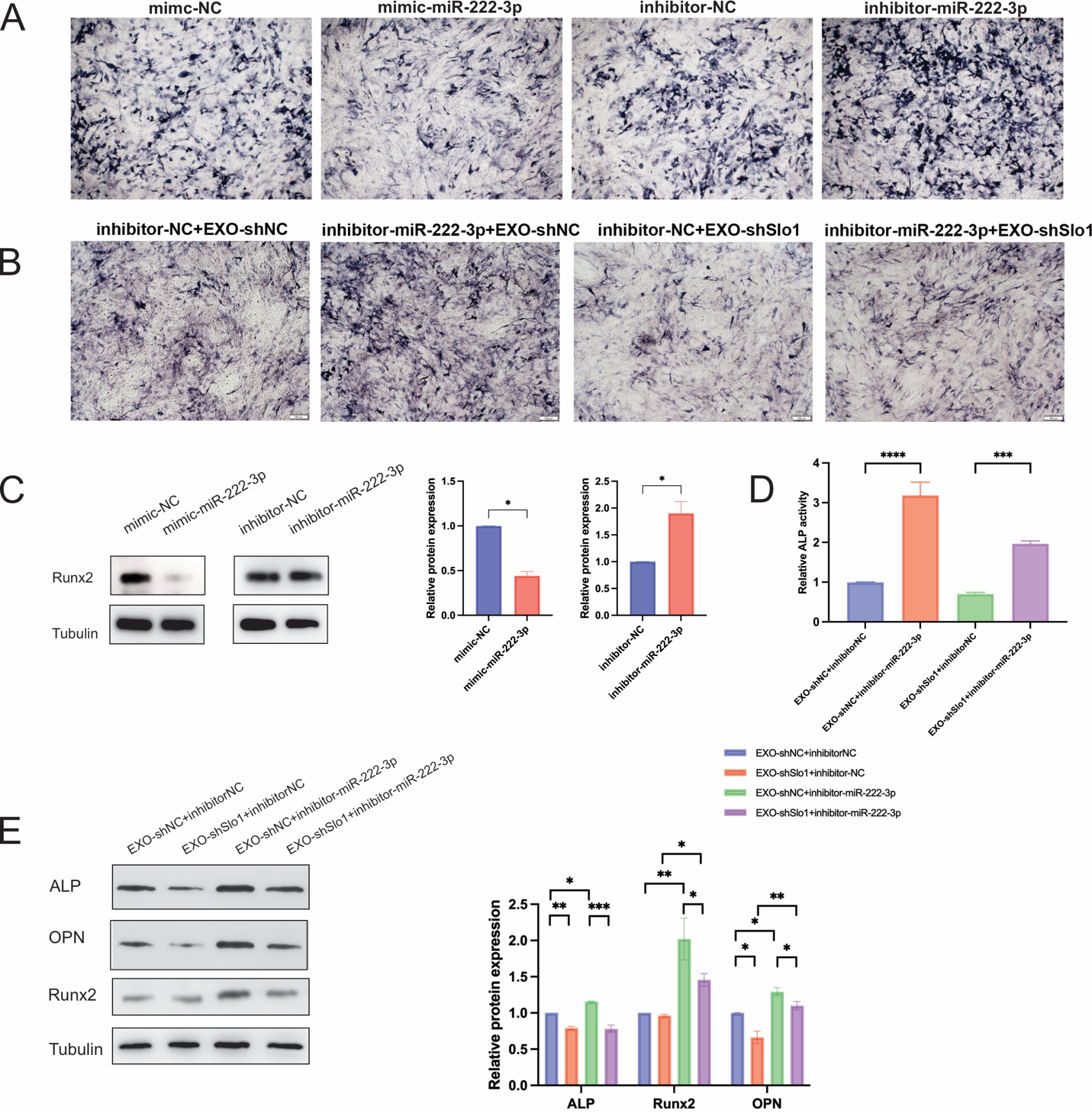


Supplementary 7. (A) The represented ALP staining images of MC3T3-E1 cells transfected with mimic and inhibitor of miR-222-3p. MC3T3-E1 cells were transfected with a NC inhibitor or miR-222-3p inhibitor, followed by treatment with EXO-shNC and EXO-shSlo1, the cells were then subjected to 7 days of osteogenic differentiation. (B) Representative images of ALP staining. (C) The protein levels of Runx2 were evaluated by WB. (D). The ALP activity of MC3T3-E1 cells transfected with a NC inhibitor or miR-222-3p inhibitor, followed by treatment with EXO-shNC and EXO-shSlo1. (E) The protein levels of ALP, OPN, and Runx2 were measured by WB. n=3, *P<0.05, **P<0.01, and ***P<0.001. (EXO-shNC means the exosomes derived from C2C12 transfected with shNC; EXO-shSlo1 means the exosomes extracted from C2C12 transfected with shSlo1).


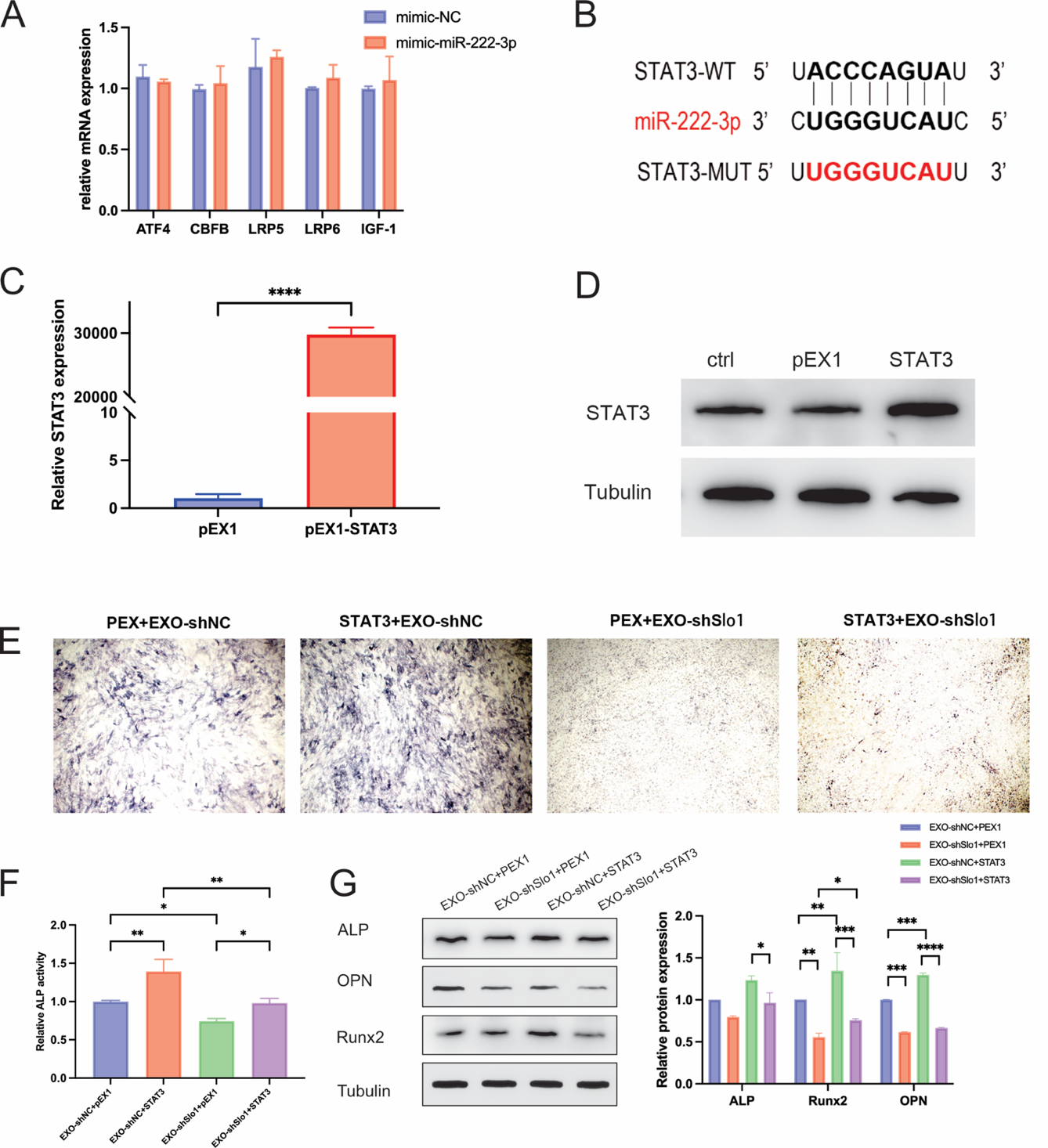


Supplementary 8. (A)Q-PCR analysis of predicted target of miR-222-3p. (B) Schematic diagram of mutant STAT3, wild-type STAT3, and miR-222-3p binding sites. (C) The mRNA expression level of STAT3 after transfection of PEX1 and STAT3. (D) STAT3 protein levels after transfection with PBS, PEX1, or STAT3. (E) MC3T3-E1 cells were transfected with a vector control, PEX1 or STAT3, followed treatment with EXO-shNC or EXO-shSlo1. The cells were then subjected to 7 days of osteogenic differentiation. Representative images of ALP staining. (F) Quantitative ALP activity was measured. (G) The protein levels of ALP, OPN, and Runx2 were evaluated by WB. n=3, *P<0.05, **P<0.01, and ***P<0.001. (EXO-shNC means the exosomes derived from C2C12 transfected with shNC; EXO-shSlo1 means the exosomes extracted from C2C12 transfected with shSlo1).
